# Genetic diversity, distribution, and structure of *Bemisia tabaci* whitefly species in potential invasion and hybridization regions of East Africa

**DOI:** 10.1371/journal.pone.0285967

**Published:** 2023-05-25

**Authors:** Hadija M. Ally, Hajar El Hamss, Christophe Simiand, M. N. Maruthi, John Colvin, Helene Delatte

**Affiliations:** 1 Université de La Réunion Site du CS 92003 97744 Cedex9, Sainte-Clotilde, Réunion, France; 2 CIRAD, UMR PVBMT, Saint Pierre, La Réunion, France; 3 Tanzania Agricultural Research Institute (TARI)–Ukiriguru Centre, Mwanza, Tanzania; 4 Natural Resources Institute (NRI), University of Greenwich, Gillingham, United Kingdom; ICAR-Central Insitute for Cotton Research, INDIA

## Abstract

Outbreaks of whitefly, *Bemisia tabaci* species in East and Central Africa, have become increasingly prevalent during the previous 25 years and are responsible for driving the spread of plant-virus diseases, such as cassava mosaic disease and cassava brown steak disease. Epidemics of these diseases have expanded their ranges over the same period, spreading from Uganda into other sub-Saharan African countries. It was hypothesised that a highly abundant ‘invader’ population of *B*. *tabaci* was responsible for spreading these diseases from Uganda to neighbouring countries and potentially hybridising with the resident cassava *B*. *tabaci* populations. Here, we test this hypothesis by investigating the molecular identities of the highly abundant cassava *B*. *tabaci* populations from their supposed origin in Uganda, to the northern, central, eastern and coastal regions of Tanzania. Partial mitochondrial cytochrome oxidase I (mtCOI) barcoding sequences and nuclear microsatellite markers were used to analyse the population genetic diversity and structure of 2734 *B*. *tabaci* collected from both countries and in different agroecological zones. The results revealed that: (i) the putative SSA1 species is structured according to countries, so differ between them. (ii) Restricted gene flow occurred between SSA1–SG3 and both other SSA1 subgroups (SG1 and SG2), even in sympatry, demonstrating strong barriers to hybridization between those genotypes. (iii) Not only *B*. *tabaci* SSA1-(SG1 and SG2) was found in highly abundant (outbreak) numbers, but *B*. *tabaci* SSA1-SG3 and the Indian Ocean (IO) species were also recorded in high numbers in several sites in Tanzania. (iv) The SSA1-(SG1 and SG2) species was distributed in both countries, but in Tanzania, the *B*. *tabaci* IO and SSA1–SG3 species predominated. These data confirm that multiple, local Tanzanian *B*. *tabaci* species produce highly abundant populations, independent of the spread of the putative invasive *B*. *tabaci* SSA1-(SG1 and SG2) populations.

## Introduction

Population outbreaks, which are characterized by rapid changes in population density from generation to generation, may occur following the introduction of a new species to an area or growth of native populations under favourable conditions [1]. Changes in population density may occur within a growing season or over a period of years [2] and may be triggered by climatic conditions, host-range usage shift and natural enemies [1]. Consequences of effects of insect outbreaks may be severe, such as the spread of diseases in humans (e.g. mosquito-vectored malaria and dengue) or plants (e.g. pathogens transmitted by insects or by direct damage) [3–5].

The group of more than 40 cryptic, whitefly species [6–9], currently still known as *Bemisia tabaci* (Hemiptera: Aleyrodidae) contains some of the most economically devastating direct pests and plant-virus vectors [10, 11]. *B*. *tabaci* species are distributed across the tropics and subtropics [12], and some of the most ancient, as determined by partial mitochondrial cytochrome oxidase I (mtCOI) barcoding sequencing, originated in sub-Saharan Africa [6–8, 13].

The *B*. *tabaci* species identified from sub-Saharan Africa (SSA), include the cassava colonizing groups SSA1 to SSA5 and non-cassava colonizing groups, such as Med (Q1, Q2 and Q3), Med ASL, MEAM1, Indian Ocean (IO), East Africa 1 (EA1) and Uganda sweetpotato (Ugsp) [9, 14–21]. Recently, five new *B*. *tabaci* species were identified from Uganda (SSA9 to SSA13) [9], including two (SSA9 and SSA10) that were found predominantly on cassava.

The geographical distribution of these species is varied: SSA1 occurs throughout the SSA regions, but is dominant in East Africa [9, 16, 20–22], whereas SSA2 to SSA4 have been reported from regions of West and Central Africa [14, 21–24], and SSA5 has been found in Ivory Coast and South Africa [14, 25]. Ugsp has only been reported from Uganda [9, 18], but IO is more widely distributed from the Indian Ocean islands, through East Africa and up to Central Africa [17, 18, 20, 21, 26]. Other species, such as Med (Q1, Q2 and Q3), Med ASL and MEAM1, have also been reported from several locations [9, 18, 22, 25, 27–29]. Highly abundant populations of *B*. *tabaci* have been recorded in many regions of SSA since the late 1980s, causing major yield reductions in cassava [20, 21, 30, 31]. In many parts of East and Central Africa, the abundance of *B*. *tabaci* on cassava and other associated crops has increased by >200-fold [16, 20, 21, 31], associated with pandemics of cassava mosaic virus disease (CMD) and cassava brown streak virus disease (CBSD) in cassava growing regions [16, 18, 20, 21, 31, 32].

Re-emergence of CMD in Uganda during the late 1980s is thought to be the result of the combination of several factors, including the recombination of the *African cassava mosaic virus* (ACMV) with *East Africa cassava mosaic virus* (EACMV) that produced the EACMV-Uganda variant (EACMV–Ug) and high abundance of *B*. *tabaci* that ensured rapid spread of CMD to neighbouring countries [30, 31, 33, 34]. Meanwhile, southward expansion of the CMD epidemic front was described as a consequence of highly abundant whitefly along south-north transect in Uganda, which was associated with whitefly migration [34].

An improved understanding of *B*. *tabaci* species’ distributions, abundances and genetic diversities in East Africa is important for the selection and targeting of appropriate management control measures. Here, we tested the hypothesis that the highly abundant populations of *B*. *tabaci* observed on cassava in Tanzania resulted from an invasion by a new species, or populations originating from the first outbreaking populations observed in Uganda. We also investigated the associated hypothesis that the ‘putative invasive’ and resident *B*. *tabaci* populations had hybridized, by using population genetics tools to examine a wide range of populations from different sites and agroecological zones in selected regions of Tanzania and Uganda.

## Materials and methods

### Study collection areas

The Tanzania Agricultural Research Institute (TARI) and Ugandan National Agricultural Research Organisation (NARO) granted the permissions for whitefly collections. In mainland Tanzania, collections were made in Arusha, Manyara, Dodoma, Morogoro, Pwani and Dar es Salaam regions, as well as in Mjini Magharibi and Unguja Kusini areas of Zanzibar Island. The Ugandan collections were from the Central region, which is characterised by tropical savannah. Tanzania, however, is characterised by a tropical climate with different climatic zones. Arusha, Manyara and Dodoma are classified as semiarid regions with an unimodal rainfall pattern resulting in a rainy season between December and April [35]. Morogoro is mid-altitude sub-humid and has a bimodal rainfall pattern, with lower rainfall (vuli) between October to December and heavy rainfall (masika) between March and May. Pwani, Dar es Salaam and Zanzibar are hot humid coastal regions, which experience bimodal rainfall patterns similar to Morogoro. Elevation in sampled area of Tanzania ranged from sea level to 1415 m above sea level (asl). In contrast to the central region in Uganda, which is characterized by the Lake Victoria crescent, the estimated annual rainfall is 952 mm driven by a bimodal pattern, where there is a rainy season from March to May and a shorter season from September to November [36]. Altitude in the Ugandan central region is around 950 to 1279 m asl.

### Sample collection

**Fig 1:**
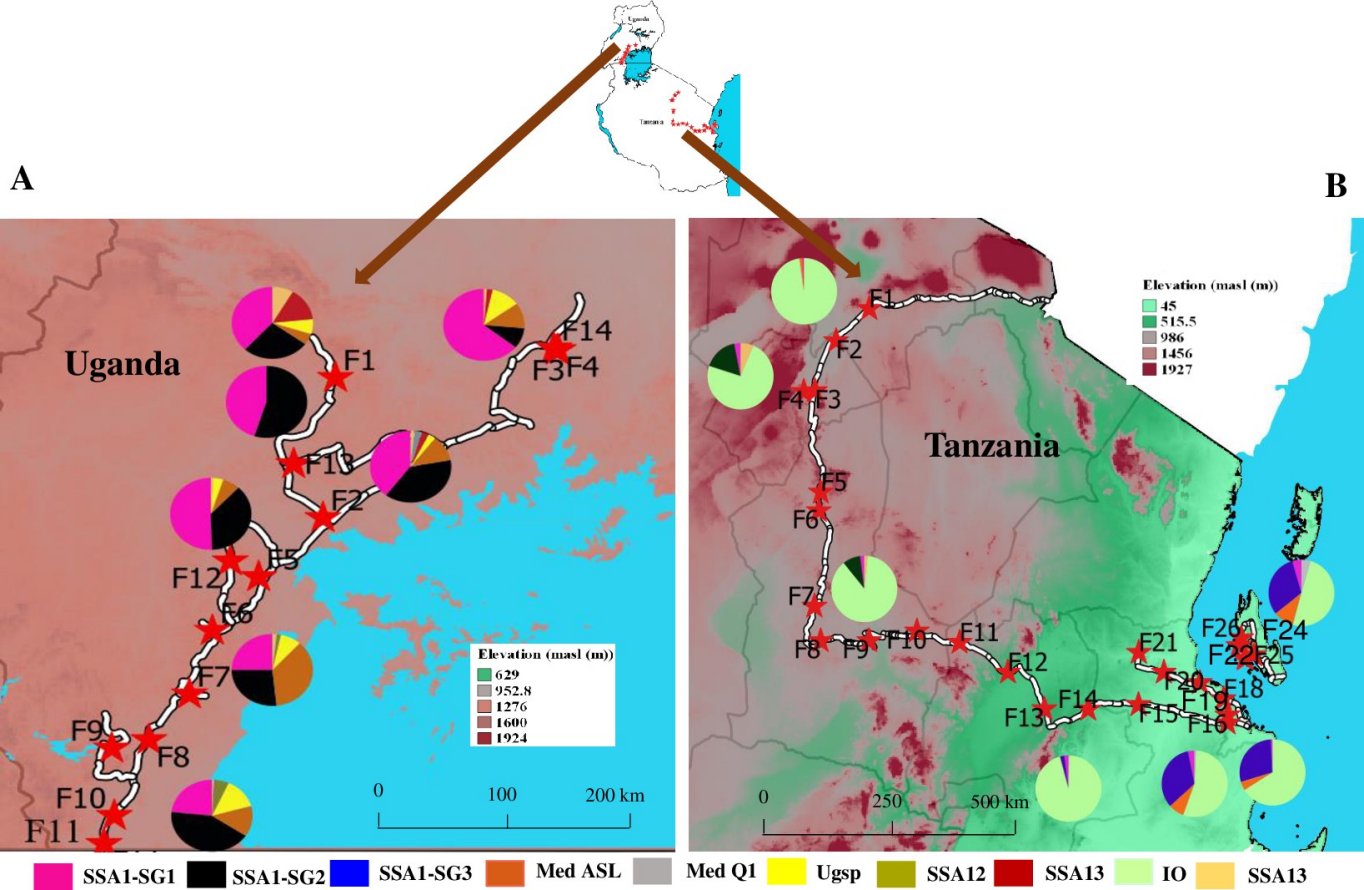
Sampling sites and composition of *B*. *tabaci* genotypes collected from agroecological zones along an elevation gradient in (A) Uganda and (B) Tanzania. The map was generated by QGIS v.2.18.

Surveys of mixed cropping systems were conducted from the 20^th^ to 28^th^ of February 2016 in Tanzania and 8^th^ to the 13^th^ of February 2017 in Uganda. Samples of *B*. *tabaci* specimens were collected from 27 fields distributed across Tanzania in the northern (Arusha and Manyara), central (Dodoma), eastern (Morogoro), and coastal mainland regions (Pwani and Dar es Salaam), as well as from Zanzibar Island (Unguja Kusini) (S1A Table). In Uganda, samples were collected from 14 fields distributed across seven districts in the Central region (Mityana, Mpigi, Wakiso, Kalungu, Masaka, Rakai and Gomba) (S1B Table). Adult whiteflies were collected from all available plant species, including weeds within and up to a distance of 5–10 m from cassava fields. Sample collection sites were spaced along the main roads at <50 km intervals, depending on the availability of cassava fields. A GPS device (Garmin © eTrex) was used to record field coordinates and a map of sample positions was created in QGIS 2.18 (https://qgis.org; (Fig 1)).

Up to 50 adult whitefly were collected for each host-plant species in each field, which included cassava (*Manihot esculenta*) as a main crop and minor crops of tomato (*Solanum lycopersicum*), eggplant (*Solanum melongena*), pumpkin (*Cucurbita moschata*), okra (*Abelmoschus esculentus*), watermelon (*Citrullus lanatus*), sweetpotato (*Ipomoea batata*), cowpea (*Vigna unguiculata*), green gram (*Vigna radiata*), cabbage (*Brassica oleracea*), groundnut (*Arachis hypogaea*), zucchini (*Cucurbita pepo*), sunflower (*Helianthus annuus*), beans (*Phaseolus vulgaris*) and weed species in the Malvaceae, Cleomaceae, Asteraceae, Solanaceae, Lamiaceae, Euphhobiaceae, Nyctaginaceae and Brassicaceae (S1 Table). Adults were collected using a mouth aspirator and then stored in microfuge tubes containing absolute ethanol prior to laboratory analysis; each sample tube, therefore, contained whitefly specimens collected from a particular site and host-plant combination.

### *B*. *tabaci* abundance, CMD and CBSD severity

Five cassava plants were randomly selected per field and the abundance of *B*. *tabaci* adults on the five uppermost leaves of each plant was counted, following the methods described in Sseruwagi *et al*. [37]. Abundance was classified as either “low”, where all host plants within a field hosted <100 adults, or “super abundant”, where at least one plant from the five randomly selected plants per field hosted >100 adult female *B*. *tabaci*. Silver leaf symptoms on pumpkin (*C*. *moschata*) were recorded when *B*. *tabaci* adults were present, and visual CMD and CBSD symptoms on cassava were scored using a scale of 1–5, where 1 indicated no disease symptoms and 5 indicated severe symptoms, as described by Mahungu *et al*. [38].

### DNA extraction

A 100x stereomicroscope (MZ8, Leica Microsystems, Nanterre, France) was used to select 20 adult *B*. *tabaci* females from each sample whenever possible (some samples had <20 females). In total, 2161 and 754 *B*. *tabaci* females from Tanzania and Uganda, respectively, were selected and DNA extraction was performed for each individual as described by Ally *et al*. [44].

### mtCOI PCR amplification and sequencing for species identification

PCR for partial mtCOI fragment amplification was conducted using a primer pair described by Mugerwa *et al*. [9]. The PCR reaction mixture was conducted in a final volume of 20 μl, containing 10 μl of type-it (2x) (Qiagen, France), 7 μl of pure HPLC water (Chromasolv, Sigma-Aldrich), 1 μl of each primer (forward and reverse) and 1 μl of DNA template. Initial denaturation of template DNA was performed at 95°C for 15 min, followed by 40 cycles of denaturation at 95°C for 30 s, primer annealing at 52°C for 45 s and extension at 72°C for 1 min; a final extension was run at 72°C for 10 min. Amplified products were visualized using QIAxcel (Qiagen, France) prior to sequencing at Macrogen, Europe©.

### Sequences analysis

Sequences were manually edited and aligned using Geneious R10 v.10.2.3 [39], and the number and distribution of haplotypes within surveyed fields was analysed using DnaSP6 Rozas *et al*. [40]. All unique haplotypes were selected and aligned using reference sequences from GenBank using ClustalW [41] within Geneious (R10 v.10.2.3) [39]. The optimum model of nucleotide substitution was selected using Jmodeltest v.2.1.10 [42]. MrBayes [43] was used to construct a phylogenetic tree using a GTR+I+G substitution model that was the optimal model identified in Jmodeltest. The analysis was run with 1,000,000 iterations of MCMC (the first 100,000 iterations were discarded) and sampled trees were made every 200 iterations, using four heated chains using MrBayes.

### Microsatellite genotyping

A set of 13 microsatellite loci was used with different repeat motifs developed for *B*. *tabaci* genotypes [44–47] (S2 Table). Three multiplex fluorescent labelled primer mixes were prepared, where the first contained Ms145, P59, P7 and WF2HO6, the second contained P62WF1GO3, WF1DO4 and P5 and the third contained CIRSSA2, CIRSSA6, CIRSSA7, CIRSSA13 and CIRSSA41. Preparation of PCR mixes and their reactions followed the methodology described above and the peaks were visualized using Gene mapper v 4.0.

### Nuclear analysis

Data were checked with MICROCHECKER software for scoring error [48]. Population genetic diversity indices were calculated within species, with a minimum number of individuals of n ≥ 5 per field. The two SSA1 subgroups, SG1 and G2, belong to the same biological species [24, 44], so they were merged under the SSA1 species for further analyses. Population genetic parameters, which comprised expected heterozygosity (He), heterozygosity calculated without biased (Hn.b), observed heterozygosity (Ho) and mean number of allele per population, were analysed following the method reported by Nei [49] in GENETIX v.4.05.2. Genetic diversity among populations (Fis) was analysed using the method utilized by Weir and Cockerham [50]. The Hardy-Weinberg equilibrium (probability test) was tested using ALREQUIN v.3.5.2.2. [51] following a method described by Guo and Thompson [52]. Allelic richness was analysed in FSTAT v.2.9.3.2 [53] using the rarefaction method. The proportion (%) of null alleles was estimated using Brookfield’s method [54] and correlations between genetic differentiation (FST/(1-FST) between *B*. *tabaci* populations within each species and different geographic distances between sampling locations were explored using the Isolde program in the online software GENEPOP [55].

Genetic structuring between populations was evaluated using STRUCTURE v.2.3.4 software [56] that assigns an individual to different genetic clusters of an unknown population, K [56]. The structure output is presented by a bar plot of posterior probability for each individual according to its genetic cluster assignation. Structure was run 10 times with an initial 10^5^ burning iterations, followed by 10^6^ MCMC iterations of potential K ranging from 1–20. Optimum K (s) were analysed using the Δk method [57] and structure output was visualized using STRUCTURE HARVESTER [58]. The software CLUMPP [59] was used for averaging the best K assignments with Bayesian probability; then, DISTRUCT was used to reconstruct the averaged bar plots obtained using CLUMPP [60] through the online program CLUMPAK [61]. Discriminant analysis of principle components (DAPC) using R v 3.4.2 software [62] with the Adegenet package [63] was used to explore genetic differentiation between populations.

Data were split into separate subsets according to species identified by mtCOI and location (Tanzania and Uganda) for analysis and comprised all SSA1 individuals sampled from both countries (including all subgroups of SSA1 identified by mtCOI barcoding analysis), SSA1 individuals collected from Tanzania and all remaining identified genotypes, excluding SSA1 (IO, Med Q1, Med ASL, Uganda sweetpotato, SSA12 and SSA13). Subsequent runs of STRUCTURE were conducted to understand substructures in, (i) SSA1–SG3 and IO from Tanzania, (ii) SSA12 and SS13 from Uganda and (iii) Med Q1 and Med ASL from both countries.

## Results

### *B*. *tabaci* identification and distribution

Partial mtCOI sequences were successfully amplified for 2734 individuals from the initial 2915 adult females collected. Those sequences were collected from different agroecological zones along an elevation gradient: in Uganda elevation ranged from 952.8 to 1276 m asl (Fig 1A) and from 45 to 1927 m asl in Tanzania (Fig 1B).

Sequence quality and length varied, with length ranged from 300 to 595 nt. We obtained sequences from 300 nt (*n =* 163), 350 nt (*n =* 200), 400 nt (*n =* 700), 500 nt (*n =* 600) to 595 nt (*n =* 1071). There were 15 mitochondrial genetic groups of *B*. *tabaci* found throughout the selected regions of Tanzania and Uganda: SSA1 (comprised SG1, *n =* 302; SG2, *n =* 287; and SG3, *n =* 234), SSA11 (*n =* 3), SSA12 (*n =* 15), SSA13 (*n =* 14), IO (*n =* 1535), Med Q1 (*n =* 17), Med ASL (*n =* 153), Ugsp (*n =* 60), Uganda 1 (*n* = 6), EA1 (*n =* 2), Sudan II (SII) (*n =* 1) and four unidentified groups (*n =* 14). The whitefly species, *Bemisia afer* (*n =* 90) was identified but excluded from further analysis. These mtCOI sequences were then used as species tags in the nuclear analysis and long (595 nt) sequences were used to reconstruct a phylogenetic tree.

**Fig 2:**
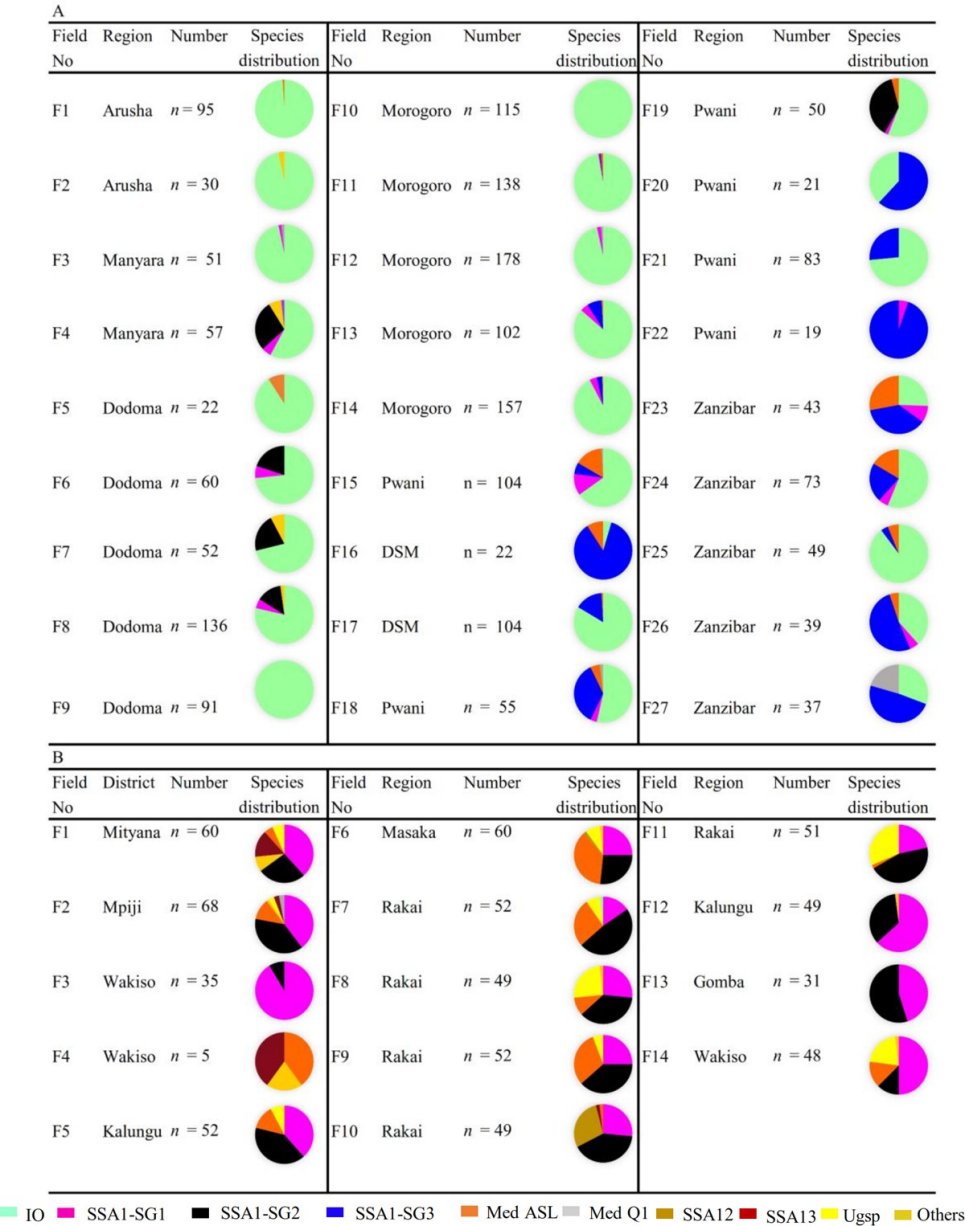
Composition of *B*. *tabaci* species per field in Tanzania (*n* = 1983) (A) and Uganda (*n* = 661) (B).

Species composition varied across agroecological zones and countries. SSA1 (SG1 and SG2) (*n* = 473, 17.3%) was the dominant species in the Central region of Uganda (Fig 1A) and was widely distributed in all but one field. All three subgroups of SSA1 (SG1 to SG3) were observed in Tanzania, albeit with significant geographical variation. SSA1–SG2 (*n* = 58, 2.12%) dominated in the highland (> 900 m asl) area of the northern part, SSA1–SG1 (*n* = 58, 2.12%) was widely distributed across the country from the low to highland and SSA1–SG3 (*n* = 234, 8.56%) was restricted to the lowland area, below 45 m asl, in Zanzibar and up to 500 m in Morogoro and Pwani regions (Fig 1B). IO (*n* = 1535, 56.14%) was the dominant species sampled in Tanzania, recorded from 26 of 27 fields (Fig 2A); observed in all agroecological zones while a single IO female (0.04%) was recorded from Uganda. Greater abundance of Med was recorded in Uganda (Fig 2B) (Med ASL: *n* = 86, 3.15%, 11 of 14 fields; Med Q1, *n* = 2, 0.07%, 1 of 14 fields) than in Tanzania (Med ASL: *n* = 67, 2.45%, 10 of 27 fields, Med Q: *n* = 15, 0.55%, 2 of 27 fields). Both Med species in Uganda were recorded in similar agroecological areas, in contrast to Tanzania where this species was observed in coastal areas. Med ASL was more frequent in Pwani and Zanzibar, while Med Q1 was dominant in Zanzibar. SSA12 (*n* = 15, 0.55%, 2 fields), SSA13 (*n* = 14, 0.51%, 4 fields) and Ugsp (*n* = 60, 2.19%, 10 fields) were recorded only in highland areas of Uganda.

There were an additional eight species recorded from different agroecological zones in Tanzania (*n* = 14) and Uganda (*n =* 4), comprising SSA11 (*n* = 3, 0.11%) and Uganda I (*n* = 6, 0.22%) in Uganda and Sudan II (*n* = 1, 0.04%) and four yet unidentified species (*n* = 14, 0.51%) in Tanzania, with EA1 (*n* = 2, 0.07%) recorded from both countries. All these were recorded from the highland area.

### Phylogenetic analysis of whitefly genetic groups

**Fig 3:**
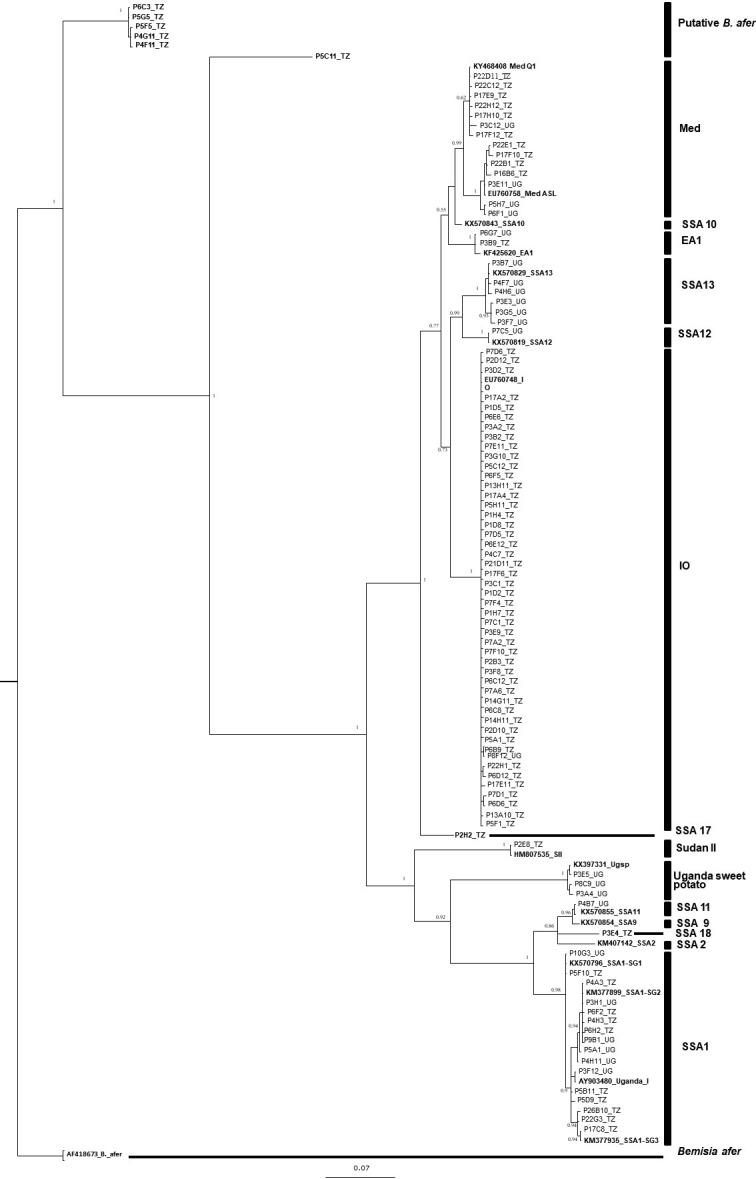
Phylogenetic tree generated using 102 mtCOI sequences selected from a total 1071 long sequences, with 595 base pair (bp) obtained from adult *B*. *tabaci* (cassava and non-cassava) and 14 reference sequences from GenBank. Sequences in bold represent references from GenBank and the four new identified groups (>4% of nucleotide identity from the closest *B*. *tabaci* sequence).

Phylogenetic analysis was carried out using long mtCOI sequences (595 nt) from adult female whitefly (*n* = 1071) sampled from Tanzania *n* = 731 and Uganda *n* = 340. We recorded 96 haplotypes, with accession numbers from MN709400 to MN709496 (S3 Table), that were used together with reference sequences from GenBank, to generate a phylogenetic tree (Fig 3). The greatest number of haplotypes (*n* = 47) was found for the IO species (*n* = 571) that apart from one were collected in Tanzania and shared 100% identity with the P6B9_TZ haplotype from Tanzania. There were two dominant haplotypes within the IO species: the first group contained 330 individuals (57.9%) and shared 100% similarity with EU76074 identified from Reunion Island [29], and the second (27.2%, *n* = 155) shared 100% identity with AY903523 reported from Uganda [19].

We found 13 haplotypes from 405 individuals of the SSA1 species; eight were recorded from Tanzania, three from Uganda, and two were common to the two countries. The majority of SSA1 comprised SSA1–SG2 (*n* = 161, 39.8%) and shared 100% with KM377899 reported in Malawi and Uganda [23]. SSA1–SG1 was the next most common, containing 153 individuals (37.8%) which shared 100% identity with KX570796 that originated from Uganda [9]. The last subgroup identified was SSA1–SG3 (*n* = 55, 13.6%), which shared 100% nucleotide identity with KM377902 described from Malawi [23] (Fig 3).

Within Med species, 14 haplotypes (*n* = 41) were found, four from Uganda and ten from Tanzania, and they clustered into two subgroups. One group contained individuals *n* = 33 sharing 99.3–100% nucleotide identity with MH205754 known as Med Africa silver leafing species (Med ASL) [64], whereas, the second group consisted of *n* = 8 individuals sharing 99–100% nucleotide identity identified as Med Q1 from MH205752 [64].

The Ugsp species contained three haplotypes (*n* = 21), which shared 99.5–100% nucleotide identity with KX397331 reported in Uganda [65]. Twelve individuals belonging to four different haplotypes, one shared 94.3% nucleotide identity with KX570843 described as SSA13 from Uganda [9] here proposed to be designated SSA17, the second haplotype shared 94.8% nt similarity to an unpublished sequence called SSA16 from Uganda, so is proposed here to be named SSA18. The remaining two other haplotypes *n* = 10 shared 79.1–85.1% nucleotide identity with *B*. *afer*, so were classified as only putative *B*. *tabaci* species.

In addition, 11 haplotypes of the least frequently occurring species such as EA1 (*n* = 2) were observed to share 99.5% nucleotide identity with KF425620, reported by Legg *et*. *al*. [16] in Tanzania; SSA11 (*n* = 3) shared 100% identity with KX570855 from Uganda [9], SSA12 (*n* = 1) shared 100% identity with KX570819 [9] found in Uganda, SS13 (*n* = 8) shared 99.0 to 100% identity with KX570833 identified in Uganda [9], Uganda I (*n* = 6) shared 100% identity with AY903480 and Sudan II (*n* = 1) shared 100% identity with HM807535.

### Sites with superabundance of *B*. *tabaci*

There was variation in adult abundance among the 41 fields surveyed in both countries. In Tanzania 10 fields (40.7%) were categorised as having a superabundance whitefly population (>100 adults per plant) (S1A Table), in which IO and SSA1–SG3 species were dominant in five and four fields, respectively, and a single field was dominated by SSA1–SG1. These fields were encountered in the Eastern and Coastal area with an elevation ranging from 500 to 45 m asl.

In Uganda, 61.5% (*n* = 8) of the fields surveyed contained superabundant whitefly populations (S1B Table). The SSA1–SG1 and SSA1–SG2 dominated in superabundant fields (99.2%); other species including Uganda 1: 0.4%; and, Med ASL: 0.4% were also observed. These fields were distributed across all agroecological zones >900 m asl, from the central to southern region that borders Tanzania (Fig 1A).

### Silver leafing symptoms

Of all 27 fields surveyed in Tanzania, pumpkin was grown in ten, amongst which, six in Dodoma, Morogoro and Dar es Salaam regions contained plants with silver leafing symptoms (S1A Table). IO was the only species observed from all samples derived from symptomatic pumpkins.

### Nuclear genetic diversity

A total of 2728 samples were successfully genotyped at 13 microsatellite loci (Tanzania: *n* = 1956; Uganda: *n* = 639). The analysis excluded 133 (4.9%) individuals that had >30.0% missing data, 117 individuals from *B*. *afer*, and low frequency species. Average allelic richness between species were moderate to high, with a range of 1.61 (SSA1–SG1 to SG3 from Tanzania) to 5.89 (SSA1–SG1 and SG2 from Uganda). We found lower observed heterozygosity (Ho) across all species than that expected (He) and the average F_IS_ per species ranged from 0.16 to 0.34 for overall populations. All populations were at the Hardy–Weinberg equilibrium, similarly no linkage disequilibrium was observed (Table 1).

**Table 1:** Population genetic diversity indices for *B*. *tabaci* sampled from Tanzania and Uganda.

Tanzania	N	Mean	Ar	Ho	Hnb	He	Fis
**IO species**							
TZ F1	104	11.17	3.3	0.42(0.22)	0.56(0.24)	0.56(0.24)	0.26
TZ F2	37	7.92	3.28	0.4(0.21)	0.56(0.25)	0.55(0.24)	0.28
TZ F3	48	7.75	3.24	0.46(0.25)	0.56(0.24)	0.55(0.24)	0.18
TZ F4	34	7.17	3.21	0.46(0.25)	0.56(0.24)	0.55(0.23)	0.19
TZ F5	16	5.5	3.27	0.48(0.26)	0.58(0.21)	0.56(0.2)	0.17
TZ F6	44	7.42	3.25	0.44(0.26)	0.56(0.26)	0.55(0.26)	0.22
TZ F7	37	6.92	3.07	0.37(0.26)	0.52(0.26)	0.52(0.25)	0.29
TZ F8	105	10.58	3.25	0.39(0.22)	0.57(0.23)	0.56(0.23)	0.32
TZ F9	98	9.83	3.15	0.45(0.25)	0.54(0.23)	0.53(0.23)	0.17
TZ F10	120	11.08	3.33	0.42(0.25)	0.58(0.25)	0.58(0.25)	0.28
TZ F11	139	11.17	3.32	0.42(0.24)	0.57(0.24)	0.57(0.23)	0.26
TZ F12	83	10.17	3.32	0.44(0.22)	0.57(0.25)	0.57(0.25)	0.23
TZ F13	91	10	3.21	0.37(0.19)	0.56(0.22)	0.56(0.22)	0.35
TZ F14	156	12.08	3.29	0.41(0.24)	0.56(0.26)	0.56(0.26)	0.28
TZ F15	71	8.67	3.27	0.36(0.23)	0.56(0.28)	0.55(0.28)	0.35
TZ F16	4	3.33	3.33	–	–	–	–
TZ F17	71	10.42	3.57	0.44(0.21)	0.63(0.2)	0.62(0.2)	0.31
TZ F18	30	8.75	3.63	0.42(0.24)	0.62(0.23)	0.61(0.22)	0.34
TZ F19	30	7.08	3.28	0.36(0.19)	0.58(0.23)	0.57(0.23)	0.38
TZ F20	10	4.17	2.95	0.36(0.24)	0.52(0.26)	0.5(0.25)	0.32
TZ F21	48	8.25	3.24	0.36(0.21)	0.55(0.25)	0.55(0.25)	0.35
TZ F23	11	4.58	3.21	0.41(0.26)	0.57(0.27)	0.54(0.26)	0.29
TZ F24	43	8.5	3.24	0.39(0.22)	0.54(0.27)	0.54(0.27)	0.28
TZ F25	40	7.17	3.11	0.38(0.2)	0.53(0.24)	0.53(0.24)	0.3
TZ F26	15	5.25	3.27	0.42(0.16)	0.56(0.23)	0.54(0.22)	0.26
TZ F27	5	3.25	3.06	0.31(0.24)	0.49(0.38)	0.43(0.34)	0.4
Grand mean	57.31	8.01	3.26	0.41(0.23)	0.56(0.25)	0.55(0.23)	0.28
**SSA1 (SG1&SG2)**							
TZ F2	3	2.33	–	–	–	–	–
TZ F4	19	4.67	3.45	0.54(0.33)	0.59(0.19)	0.58(0.18)	0.1
TZ F6	15	5.42	3.94	0.52(0.23)	0.62(0.17)	0.6(0.17)	0.16
TZ F7	11	4.58	3.67	0.48(0.25)	0.57(0.18)	0.55(0.18)	0.17
TZ F8	26	5.92	3.62	0.44(0.13)	0.56(0.17)	0.55(0.16)	0.22
TZ F11	2	2.5	–	–	–	–	–
TZ F12	2	2.08	–	–	–	–	–
TZ F13	2	2.5	–	–	–	–	–
TZ F14	3	2.17	–	–	–	–	–
TZ F15	3	2.67	–	–	–	–	–
TZ F16	1	1.42	–	–	–	–	–
TZ F17	1	1.75	–	–	–	–	–
TZ F18	4	2.92	–	–	–	–	–
TZ F19	2	2.42	–	–	–	–	–
TZ 21	1	1.3	–	–	–	–	–
TZ 23	1	1.5	–	–	–	–	–
Grand mean	6	2.88	3.67	0.5(0.24)	0.59(0.18)	0.57(17)	0.16
**SSA1 (SG3)**							
TZ F13	13	4.58	1.6	0.45(0.22)	0.6(0.18)	0.57(0.18)	0.26
TZ F14	12	4.33	1.59	0.54(0.24)	0.59(0.19)	0.56(0.18)	0.08
TZ F15	18	4.92	1.63	0.54(0.25)	0.63(0.13)	0.61(0.13)	0.15
TZ F16	19	6.08	1.65	0.55(0.17)	0.65(0.14)	0.63(0.13)	0.16
TZ F17	15	5.42	1.63	0.55(0.15)	0.63(0.13)	0.6(0.13)	0.13
TZ F18	16	5.5	1.63	0.41(0.21)	0.63(0.13)	0.61(0.12)	0.36
TZ F19	14	4.67	1.58	0.41(0.2)	0.58(0.16)	0.56(0.15)	0.3
TZ F20	14	5	1.63	0.39(0.24)	0.63(0.15)	0.61(0.14)	0.4
TZ F21	12	4.58	1.61	0.43(0.21)	0.61(0.14)	0.58(0.13)	0.32
TZ F22	4	2.83	–	–	–	–	–
TZ F23	20	6.5	1.65	0.44(0.16)	0.65(0.12)	0.63(0.12)	0.32
TZ F24	5	3.17	1.59	0.44(0.28)	0.59(0.29)	0.49(0.23)	0.29
TZ F25	2	2.17	–	–	–	–	–
TZ T26	14	4.75	1.61	0.49(0.21)	0.61(0.13)	0.59(0.12)	0.21
TZ 27	14	5.08	1.58	0.51(0.23)	0.58(0.13)	0.56(0.13)	0.14
Grand mean	12.8	4.64	1.61	0.47(0.21)	0.61(0.15)	0.58(0.15)	0.24
**Med Q1**							
TZ F15	1	1.5	–	–	–	–	–
TZ F18	2	2	–	–	–	–	–
TZ F27	12	4.75	4.75	0.39(0.3)	0.56(0.22)	0.54(0.21)	0.32
Grand mean	5	2.75	4.75	0.39(0.)	0.56(0.22)	0.54(0.21)	0.32
**Med ASL**							
TZ F11	2	1.5	–	–	–	–	–
TZ F12	2	1.25	–	–	–	–	–
TZ F13	3	1.83	–	–	–	–	–
TZ F14	1	1.17	–	–	–	–	–
TZ F15	18	4	3.88	0.26(0.19)	0.42(0.3)	0.41(0.29)	0.38
TZ F16	2	1.83	–	–	–	–	–
TZ F17	3	2.75	–	–	–	–	–
TZ F18	1	1.5	–	–	–	–	–
TZ F19	2	1.83	–	–	–	–	–
TZ F23	4	2.75	–	–	–	–	–
TZ F24	19	4.42	4.11	0.36(0.26)	0.44(0.29)	0.42(0.29)	0.18
TZ F25	4	2.5	–	–	–	–	–
TZ F26	3	2.25	–	–	–	–	–
Grand mean	4.92	2.28	4	0.31(0.23)	0.43(0.3)	0.42(0.29)	0.49
**Uganda**							
**SSA1 (SG1&SG2)**							
UG F1	15	6	5.65	0.44(0.24)	0.51(0.32)	0.5(0.31)	0.17
UG F2	23	6.08	5	0.38(0.26)	0.5(0.31)	0.49(0.3)	0.24
UG F3	36	8	5.32	0.38(0.28)	0.49(0.3)	0.48(0.29)	0.22
UG F4	3	2.42	–	–	–	–	–
UG F5	41	8.58	5.77	0.41(0.28)	0.52(0.33)	0.52(0.32)	0.21
UG F6	30	8.17	5.98	0.35(0.2)	0.57(0.29)	0.56(0.28)	0.38
UG F7	36	9	6.38	0.42(0.23)	0.59(0.28)	0.58(0.27)	0.3
UG F8	34	9.67	6.26	0.4(0.24)	0.55(0.28)	0.54(0.27)	0.29
UG F9	32	8.5	5.92	0.38(0.25)	0.56(0.24)	0.56(0.23)	0.32
UG F10	35	9.5	6.61	0.39(0.22)	0.57(0.29)	0.56(0.28)	0.32
UG F11	35	9	6.43	0.37(0.23)	0.59(0.28)	0.58(0.28)	0.37
UG F12	50	10.5	6.16	0.39(0.21)	0.56(0.28)	0.56(0.27)	0.31
UG F13	35	8	5.54	0.37(0.24)	0.51(0.32)	0.5(0.31)	0.27
UG F14	35	7.83	5.54	0.38(0.25)	0.51(0.3)	0.5(0.29)	0.25
Grand mean	31.43	7.95	5.89	0.39(0.24)	0.54(0.29)	0.53(0.28)	0.28
**Med ASL**							
UG F1	4	2.92	–	–	–	–	–
UG F2	15	5.08	3.53	0.35(0.22)	0.54(0.29)	0.52(0.28)	0.36
UG F4	1	1.18	–	–	–	–	–
UG F5	11	4.17	3.31	0.41(0.3)	0.52(0.29)	0.49(0.27)	0.23
UG F6	29	7	3.56	0.31(0.24)	0.54(0.29)	0.53(0.29)	0.44
UG F7	17	5.25	3.44	0.39(0.29)	0.58(0.22)	0.56(0.21)	0.33
UG F9	17	5.5	3.52	0.46(0.33)	0.56(0.29)	0.54(0.28)	0.19
UG F11	2	2.17	–	–	–	–	–
UG F14	8	3.33	2.87	0.38(0.36)	0.43(0.33)	0.4(0.31)	0.14
Grand mean	11.56	4.07	3.37	0.38(0.29)	0.53(0.29)	0.51(0.27)	0.28
**Ugsp**							
UG F1	3	2.25	–	–	–	–	–
UG F2	2	1.64	–	–	–	–	–
UG F5	3	2.42	–	–	–	–	–
UG F6	5	3.58	2.77	0.33(0.3)	0.51(0.33)	0.45(0.3)	0.38
UG F7	3	2.5	–	–	–	–	–
UG F8	14	4.75	2.69	0.39(0.29)	0.54(0.28)	0.52(0.27)	0.29
UG F9	3	2.5	–	–	–	–	–
UG F11	15	6.33	2.92	0.3(0.28)	0.53(0.36)	0.52(0.35)	0.44
UG F14	10	5.17	2.84	0.4(0.28)	0.56(0.21)	0.53(0.2)	0.28
Grand mean	6.44	3.46	2.81	0.36(0.29)	0.54(0.3)	0.51(0.28)	0.35
**SSA12**							
UG F10	13	4.08	4.22	0.31(0.31)	0.44(0.25)	0.43(0.24)	0.31
**SSA13**							
UG F1	8	4.58	3.97	0.48(0.28)	0.64(0.27)	0.59(0.25)	0.26
UG F2	1	1.27	–	–	–	–	–
UG F10	1	1.5	–	–	–	–	–
UG F4	3	2.08	–	–	–	–	–
Grand mean	3.25	2.36	3.97	0.48(0.28)	0.64(0.27)	0.59(0.25)	0.26

*B*. *tabaci* species analysed by study site (see Fig 2 for further site description). N: individuals number within a population; Mean: average number of allele per population; Ar: allelic richness; F_IS_: is a measure of deviation from panmixia at local scales; Ho: observed heterozygosity; He: expected heterozygosity; and, Hn.b: heterozygosity calculated without biases, no deviation from Hardy–Weinberg equilibrium (P>0.05) for all populations. The population genetic diversity indices were calculated considering a minimum number of individuals of *n* > 5 per field. “–“: too low sample size to perform the analysis and numbers in bracket is standard deviation.

#### Distinct genetic clusters revealed within SSA1 species from Tanzania and Uganda

**Fig 4:**
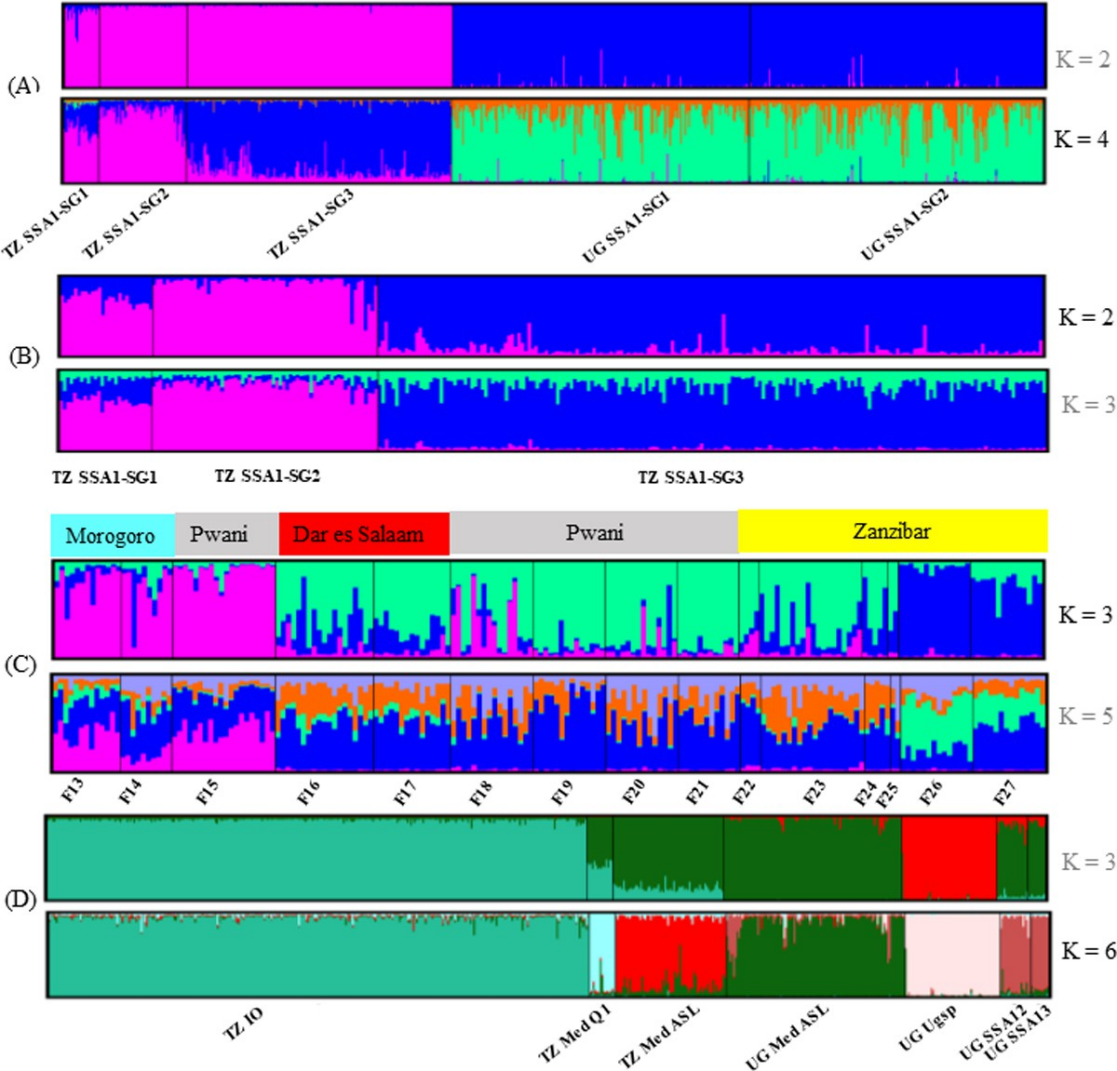
Different K populations of *B*. *tabaci* (A) SSA1 from Tanzania (TZ) and Uganda (UG), (B) SSA1 from Tanzania, (C) SSA1–SG3 from Tanzania and (D) non–cassava species. Structure bar plots are based on 12 microsatellite loci. Individuals were arranged according to mtCOI species assignment and field number separated by the black line. For each dataset, the optimal K selected by STRUCTURE HARVESTER is presented in black. DSM is Dar es Salaam.

This analysis involved the dataset containing SSA1 species including its three sampled subgroups (SG1, SG2 and SG3) from Tanzania and Uganda. A total of 729 individuals and 12 loci were used (Tanzania *n* = 288, Uganda *n* = 441). Bayesian clustering analysis separated our dataset countrywide. The best K (cluster of unknown population) for SSA1 (Tanzania *n* = 288, Uganda *n* = 441) were K = 2 and 4, using Evanno’s method [57]. A first level of differentiation was observed at K = 2, where two genetic clusters are linked to their geographic origin (country) (Fig 4A). At K = 4, the two genetic clusters were found in each country and only individuals of the SG3 collected in Tanzania clearly differed from SG1 and SG2 (Fig 4A). Similar results were found using a Discriminant Analysis of Principle Component (DAPC) analysis at K = 4 (S1 Fig). Further analysis on SSA1–SG1 and SG2 showed existence of significant isolation by distance (IBD) between countries (S2 Fig) with Mantel tests (P<0.05).

We analysed data from 288 individuals of SSA1 from Tanzania separately to understand the interactions between the genetic clusters of the three subgroups (SG1, SG2 and SG3). Two distinct genetic clusters were observed at K = 2, one dominated by SSA1 (SG1 and SG2) (Fig 4B) and the other contained individuals of SG3. No further differentiation was observed between subgroups when the number of assumed genetic clusters was increased at further K and no genetic difference was observed between SG1 and SG2 (K = 3; Fig 4B).

Individuals of SSA1–SG3 were analysed alone to understand their substructure. At K = 3, three genetic clusters were observed (Fig 4C). The first genetic cluster was dominated by individuals collected from eastern Tanzania (Morogoro and Pwani), and the subsequent clusters were dominated by individuals sampled from different sites; the remaining cluster contained individuals from Zanzibar (Fig 4C). Despite these distinct clusters, the structure pattern showed limited sharing of genetic information among a few individuals (Fig 4C).

#### Nuclear genetic diversity of non–cassava species

A subset of 582 individuals (Tanzania: *n* = 392; Uganda: *n* = 190) were used to understand the nuclear genetic diversity and potential gene flows between the different genetic groups. Based on mtCOI markers, these individuals belonged to IO (*n* = 313), Med Q1 (*n* = 17), Med ASL (*n* = 169) Uganda sweetpotato (*n* = 60), SSA12 (*n* = 12) and SSA13 (*n* = 10). Only a few individuals of IO (*n* = 313, 21%) were randomly selected from the study sites to avoid bias due to high frequency of occurrence of this species in our sampling.

The best K population was considered at K = 6 separating all species, except SSA12 and SSA13 (Fig 4D). Med ASL from Tanzania appeared to differ from the one from Uganda, although some individuals from the two countries shared similar genetic backgrounds.

The Med Q1 and Med ASL species from the two countries were analysed separately, to understand their population sub-structuring. The optimum K-value was K = 4, where the genetic clusters initially separated according to country (S3A Fig). At K = 4, the genetic clusters differentiated the two species (Med Q1, Med ASL) into four genetic clusters; two of these clusters were dominated by Med Q1 and Med ASL from Tanzania and Uganda Med ASL was further sub-structured. Despite these well-defined structures, some admixture was noticed between Tanzania and Uganda Med ASL populations, sharing genetic background between a few individuals (S3A Fig). Results on IBD showed a strong and significant correlation between geographic and genetic distances (Mantel test, P<0.05) between populations of Med ASL, forming two clusters, and each linked to a country (S4 Fig). No further analysis could be performed on Med Q1 due to the low number of individuals sampled. SSA12 and SSA13 populations were also analysed to further understand population structure. The optimum K-value was K = 2, with each genetic cluster separating the two species well. There was some indication of a partial common genetic background (S3C Fig), however, but because these analyses were performed on so few individuals (n = 26), they need to be interpreted with caution.

Analysis of IO individuals had a K = 3 optimum, although initial differentiation began at K = 2 (S3B Fig); three sub-populations were observed at K = 3, and there was no association of the observed structure with sampling fields or host plants and no isolation by distance was observed between populations or genetic clusters (Mantel test, P > 0.05) (S5 Fig).

## Discussion

This study assessed *B*. *tabaci* species diversity, distribution and genetic structure along geographical and elevational transects from central Uganda, north and east to the coastal region of Tanzania, including the island of Zanzibar. Fifteen of the *B*. *tabaci* cryptic species were collected from a wide range of areas. Among these, IO was the most dominant in Tanzanian sites and was recorded from all agroecological zones sampled. In Uganda, SSA1 (SG1 and SG2) dominated in all sites surveyed. Two new SSA1 species were identified and putatively named SSA17 and SSA18. Nuclear analysis revealed distinct genetic clusters of SSA1 populations between the two countries with only limited gene flow between the populations of both countries.

### IO distribution, abundance genetic diversity

IO was widely distributed across all regions surveyed in Tanzania and it was the most abundant species in five of the 10 fields in which *B*. *tabaci* was classified as superabundant, demonstrating its capacity to reach outbreak levels. IO has been observed in other East and Central African countries, including Uganda, Kenya and Central African Republic, but at lower abundance [16–18, 21], and appears to also be indigenous to the south-west Indian Ocean (SWIO), including islands of Réunion, Mauritius, Madagascar, Comoros and Seychelles [46, 66]. IO has previously been reported from the northwest, central and eastern regions of Tanzania [20, 46], but found in lower abundance than reported here for the coastal area. Climatic conditions along coastal Tanzania, which are similar to those of the tropical islands of the SWIO, may favour this genotype.

The nuclear analysis performed on *B*. *tabaci* IO provided evidence of three genetic clusters but showed no link to geographical location. Similarly, there was no significant evidence of genetic isolation by distance between sites. The IO species was present across all sampling fields, with evidence of gene flow between populations of the different sampled fields, regions and even between Tanzania mainland and Zanzibar Island. This extensive distribution of IO may be facilitated by movement of horticultural crops from production sites to market. Arusha, Moshi, Mbeya, Iringa and Tanga are among the major vegetable growing regions in Tanzania [67], from which produce is supplied to markets in cities and towns, including Zanzibar.

The wide distribution of the *B*. *tabaci* IO in all agroecological zones of Tanzania may indicate the capacity of this genotype to adapt to a wide range of environmental conditions, in contrast to other species recorded in this study, such as Ugsp that was only observed in Uganda. IO belongs to the most invasive phylogenetic clade of whiteflies that also includes MEAM1 and Med [6, 46]. Although high levels of abundance of IO are currently only found in Tanzania, it is possible this species could become an invasive species in nearby Eastern African countries, so it should be monitored closely, especially because it is a vector for tomato yellow leaf curl virus [66].

We recorded 96 mitochondrial haplotypes, the majority of which (*n* = 47) were from *B*. *tabaci* IO. This is unsurprising, because 56.14% of the analysed sequences were IO. Delatte *et al*. [46] compared the whitefly diversity in the Indian Ocean islands versus mainland, using many fewer samples, and reported higher levels of diversity of IO on the mainland. Despite this level of haplotype diversity in our study, most IO species belong to two major haplotypes, sharing 100% nucleotide identity with KX397323 [65] and AY903523 [19] identified from Reunion Island and Uganda, respectively. No link was found between those haplotypes, sites and agroecological zones, the nuclear genetic clusters or host plants and the IO individuals were widely distributed from the mainland to Zanzibar. Interestingly, 60% of fields cultivated with pumpkin in Tanzania showed silver leafing symptoms that were attributed to the presence *B*. *tabaci* IO. MEAM1 and IO of the *B*. *tabaci* complex have been reported to induce this physiological damage on cucurbit species [26, 68–71].

### SSA1 species distribution, abundance and genetic diversity

The other most dominant species collected in our study was SSA1 (30.1%), represented by its three subgroups (SG1, SG2 and SG3). SSA1 (SG1 and SG2) was the most abundant group in the Central region of Uganda, comprising 70.7% of all sampled individuals and it was found across all study sites and in a wide range of agroecological zones (more so than for IO); eight fields showed superabundant populations that were attributed to this species. Several studies have reported the presence of SSA1 (SG1 and SG2) in Uganda [9, 18], Tanzania, Rwanda, Burundi, Kenya, Democratic Republic of Congo (DRC) [16, 24, 72], Malawi [22], Central African Republic [21], Cameroon [21, 24], Benin and Togo [22]. Given its wide distribution in different agroecological zones of West, Central and East Africa, this species is clearly well adapted to the SSA region.

The SSA1–SG3 differed from SSA1 (SG1 and SG2) with evidence of restricted gene flow between individuals even in sympatric sites (see Fig 4C). Moreover SSA1-SG1, -SG2 harbour a common strain of Wolbachia different from that of SG3, which also indicates restricted gene flow between them [23].

We also, found SSA1 subgroups SG1 and SG2 occurring in sympatry and the nuclear analysis revealed they fully interbreed (Fig 4A). Previous studies have reported similar findings [24, 44]. Despite the lack of genetic differentiation between both subgroups within a country, their genetic structure differed between countries, with significant isolation by distance (IBD) found between populations of both countries. This might be due to different climatic condition between the sampled sites, so confirmation of a geographical differentiation and no population movements between countries requires analysis of a greater number of samples collected nearer to each other from the Ugandan border to sites in Tanzania.

Higher allelic richness occurred within the Uganda SSA1 (SG1 and SG2) compared with the Tanzania SSA1–SG2 population. The presence of high allelic richness indicates high genetic diversity within populations [65], and high genetic diversity within SSA1 subgroups from Uganda is supported by the observed high genetic diversity (F_is_). It is possible this diversity is linked to adaptation to the local environment, which is very different from coastal Tanzania; however, we are unable to confirm this, due to the small number of SSA1 (SG1 and SG2) individuals from Tanzania compared with Uganda. Another hypothesis linked to this higher diversity within and between species in Uganda [9] could point this country and highland areas being centres of diversification of whitefly species in sub-Saharan Africa. An improved understanding of the occurrence of high *B*. *tabaci* genetic diversity in Uganda is crucial for pest management and will need further investigation, because high numbers of *B*. *tabaci* species now persist in this region [9, 18].

The remaining subgroup of SSA1 found in this survey with relatively high abundance in the coastal region of Tanzania was SSA1–SG3 (Fig 1B). This subgroup was dominant on cassava in three fields and at levels classified as superabundant, to SSA1 (SG1 and SG2) and IO reported herein. The occurrence of SSA1–SG3 in similar agroecological zones has been reported in Tanzania [20, 73] previously, as well as in Central African Republic, Malawi and DRC [16, 21, 23, 24]. The presence of SG3 in the coastal region of Tanzania in greater abundance than the other SSA1 subgroups on cassava, together with the greater occurrence of CBSD in this area [74], indicates that this subgroup may be the most likely vector spreading this disease in this area.

Three mtCOI haplotypes were found within SSA1–SG3. The major haplotype (*n* = 55) contained individuals sampled from different sites; we also, observed three distinct genetic clusters of nuclear diversity, one of which consisted of individuals (*n* = 121, 63%) collected from all the study sites (Fig 4C). The two remaining clusters were site restricted, one in Morogoro and Pwani and the other in Zanzibar. It is probable that these genetic differences reflect the different agroecological zones, i.e. the hot sub-humid condition of Morogoro, whereas Zanzibar is in the hot humid coastal zone.

### Med species distribution and genetic diversity

Two distinct populations of Med (Med ASL and Med Q1) were found that have recently been demonstrated to be two separate biological species [64]. They were found in both countries; however, only two adults of Med Q1 were recorded from Uganda. We found the Med ASL species was widely distributed across both countries; nevertheless, Med Q1 from Tanzania was restricted to the coastal zone. Previous studies reported the occurrence of Med Q1 in East Africa [16, 18, 21], and both Med ASL and Med Q1 have been found in West Africa, with a different relative distribution to this study. Med ASL was reported as the dominant species in Benin and Togo, whereas Med Q1 was dominant in Burkina Faso [28] and Senegal [27]. Med ASL has not been recorded outside of Africa and, so far, has only been reported from sub-Saharan Africa. We found this species in several agroecological zones of Tanzania and Uganda indicating its ability to occupy a diverse range of environmental conditions within sub-Saharan Africa.

In this study, we also recorded Med ASL from a wide range of agroecological zones in both countries. Med Q1 was reported to be a recent invader to South Africa [25], because it is considered as a native to the Mediterranean basin. It has been extensively reported from many countries and is one of the principal invasive whitefly species worldwide (together with the MEAM1) [21, 25]. Furthermore, the blasted Med Q1 sequences of the present study shared 100% nuclear identity with sequences from China and Italy [75, 76], so the Med Q1 is most likely to be an invasive, non-indigenous species in Tanzania and Uganda.

The nuclear analysis clearly separate Med ASL species into distinct genetic clusters and showed its genetic composition differed between the two countries with significant evidence of genetic isolation by distance between countries. Thus, the difference in genetic structure may be associated with geographical isolation between populations, due to low migration between countries; nevertheless, additional samples are required to confirm this hypothesis.

### Distribution and genetic diversity of other *B*. *tabaci* species

Similar to previous studies [9, 37], we recorded SSA11, SSA12, SSA13, Uganda 1 and Ugsp only in Uganda, indicating that some species may be specific to geographic location. In addition, new SSA species putatively named as SSA17 and SSA18 were found in Tanzania. These findings indicate the SSA region has an even greater diversity than expected; thus, more research is needed to understand this diversity and to increase understanding of the potential distribution of new species.

## Conclusions

The complexity of *B*. *tabaci* species distributions was greater than expected in the surveyed area: with the capacity of not only SSA1 (SG1 and SG2) species to induce superabundant populations, but other species, such as IO and SSA1–SG3, which also occurred at high densities. *B*. *tabaci* populations and species diversity differed between Tanzania and Uganda, indicating that the causes of population differences and outbreaks are multifactorial. Thus, we reject our hypothesis that superabundant *B*. *tabaci* species’ populations observed in the eastern region of Tanzania are linked to a recent invasion of populations from Uganda. We conclude that many different *B*. *tabaci* species have the potential to develop highly abundant populations without having experienced a novel introduction or invasion of a new population or species into their region. In addition, the minimal gene flow exhibited between SSA1–SG3 with SSA1 (SG1 and SG2) provides further evidence that these are different biological species [77]. Further, abundance of *B*. *tabaci* is clearly a result of a combination of factors, including ecological niche, climatic conditions, virus presence/absence and cassava genotype. Further research into understanding their effects on *B*. *tabaci* abundance is required and this should concentrate on the development of cassava varieties not only possessing resistances to both viruses, but also on resistance to the *B*. *tabaci* species present in the various agroecological zones.

## Supporting information

S1 Fig DAPC analysis performed at K = 4 on 729 individuals of SSA1 species (n _Tanzania_ = 288, n _Uganda_ = and 441). Each cluster represents the dominant individuals within SSA1 species.
(TIF)

S2 Fig Isolation by distance graph comparing geographic Euclidian distances (Km) and genetic distances (FST/(1-FST) of Tanzania and Uganda SSA1–SG1/SG2 populations. (TIF)

S3 Fig Different population structures bar plot of B. tabaci (A) Med from Tanzania and Uganda (B) IO (Tanzania) (C) SSA12 and SSA13 from Uganda. Individuals were arranged according to mtCOI per site but due to few individuals observed per site for Med and SSA12 and SSA13 they were merged but for IO, it was presented per site. Black line separated populations. For each data set the optimal K was selected using STRUCTURE HARVESTER.
(TIFF)

S4 Fig Isolation by distance graph comparing geographic Euclidian distances (Km) and genetic distances (FST/(1-FST) of Tanzania and Uganda Med ASL populations. (TIF)

S5 Fig Isolation by distance graph comparing geographic Euclidian distances (Km) and genetic distances (FST/(1-FST) of Tanzania IO populations. (TIF)

S1 Table Host plants and location of sampled adult B. tabaci and evidence of disease on cassava in A) Tanzania and B) Uganda.
(ZIP)

S2 Table Characteristics of microsatellite loci used in the nuclear analysis. (DOCX)

S3 Table *B*. *tabaci* haplotypes and their distribution in Tanzania and Uganda. (DOCX)
