# Supplementary figures and images for: Genetic diversity, distribution, and structure of Bemisia tabaci whitefly species in potential invasion and hybridization regions of East Africa
Source: PLoS One. 2023 May 25;18(5):e0285967. doi: 10.1371/journal.pone.0285967 (PMC10212157; doi:10.1371/journal.pone.0285967)

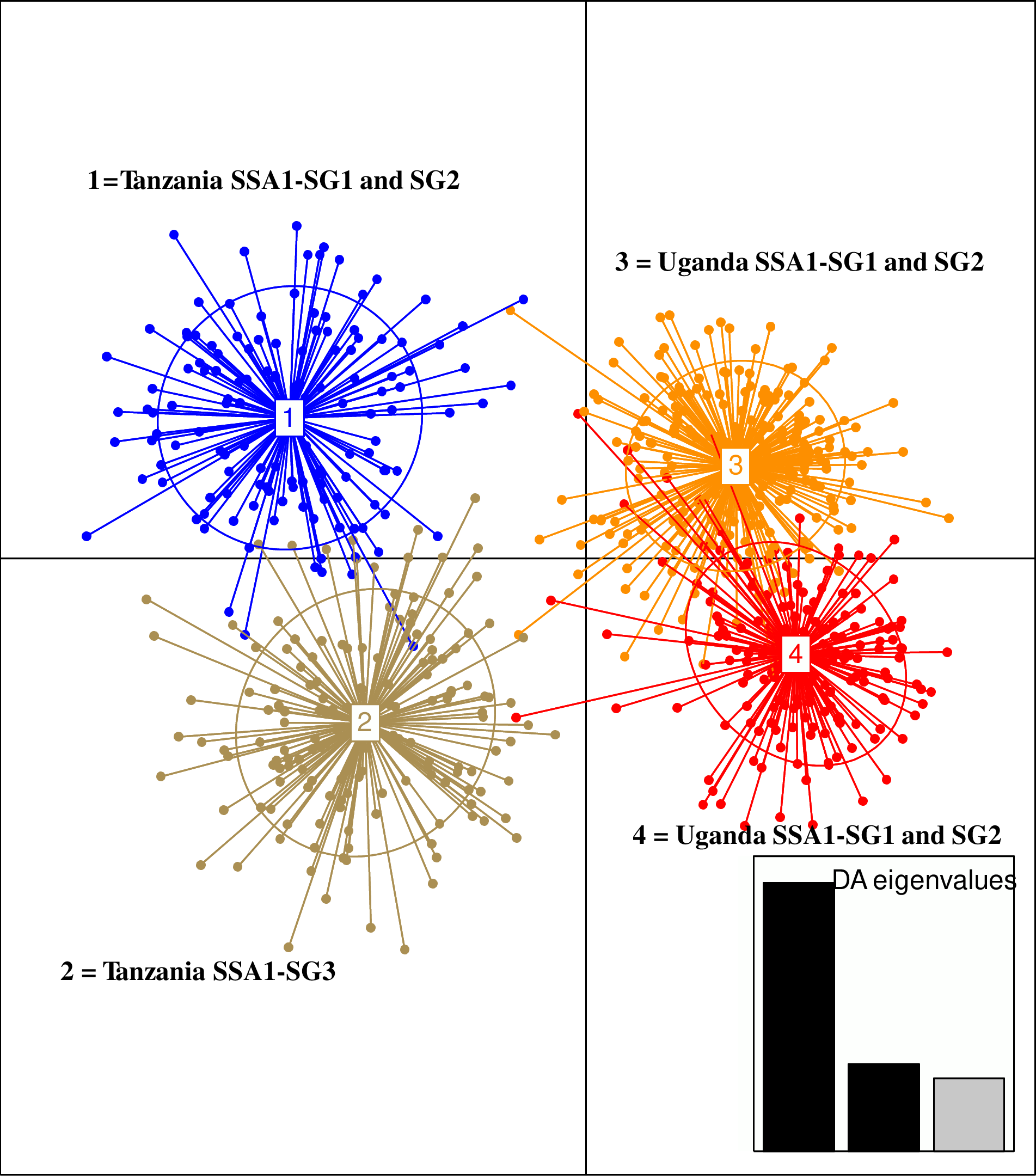

Supplement: S1 Fig — Each cluster represents the dominant individuals within SSA1 species. (TIF) [file pone.0285967.s001.tif]

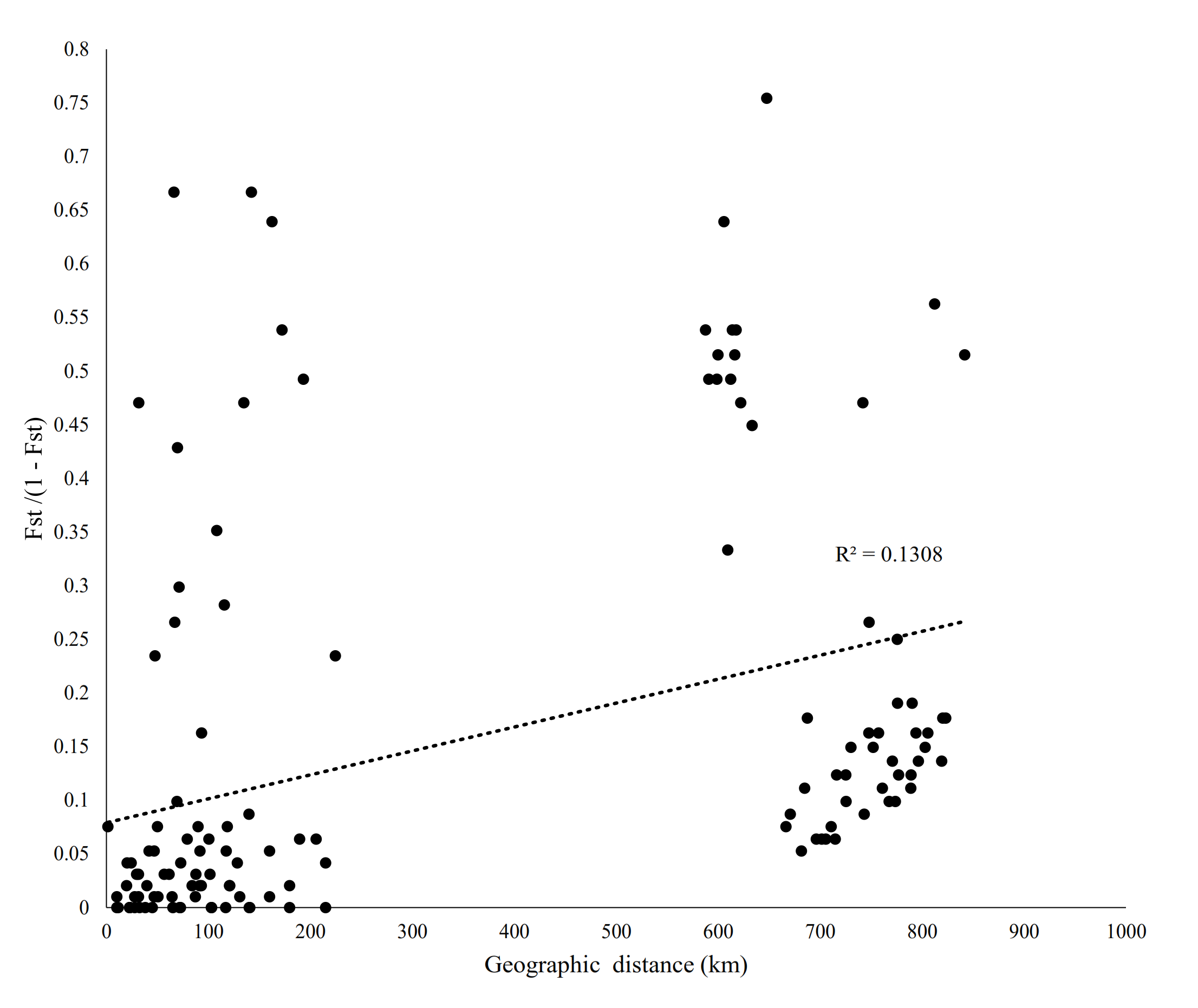

Supplement: S2 Fig — (TIF) [file pone.0285967.s002.tif]

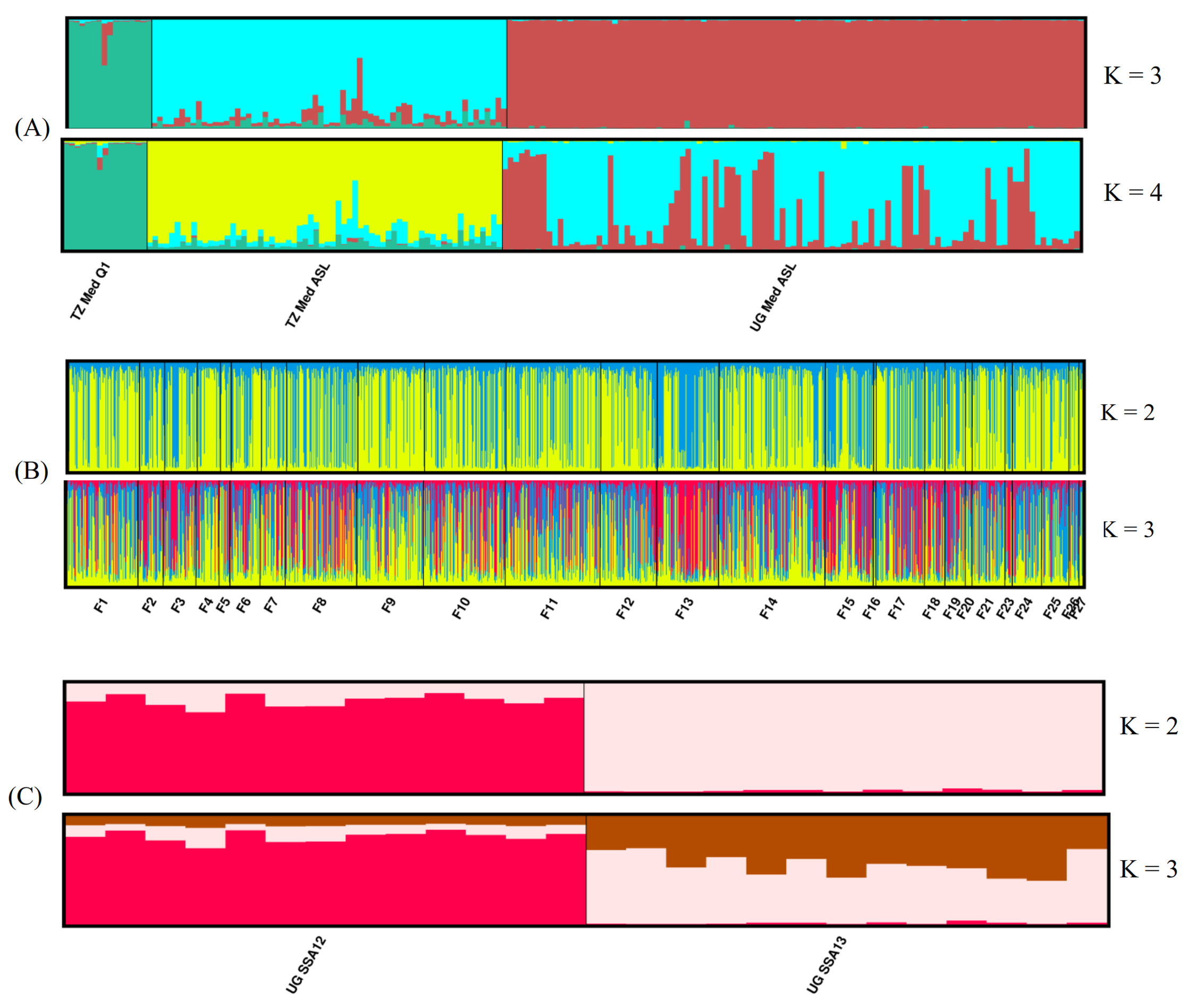

Supplement: S3 Fig — Different population structures bar plot of B. tabaci (A) Med from Tanzania and Uganda (B) IO (Tanzania) (C) SSA12 and SSA13 from Uganda. Individuals were arranged according to mtCOI per site but due to few individuals observed per site for Med and SSA12 and SSA13 they were merged but for IO, it was presented per site. Black line separated populations. For each data set the optimal K was selected using STRUCTURE HARVESTER. (TIFF) [file pone.0285967.s003.tiff]

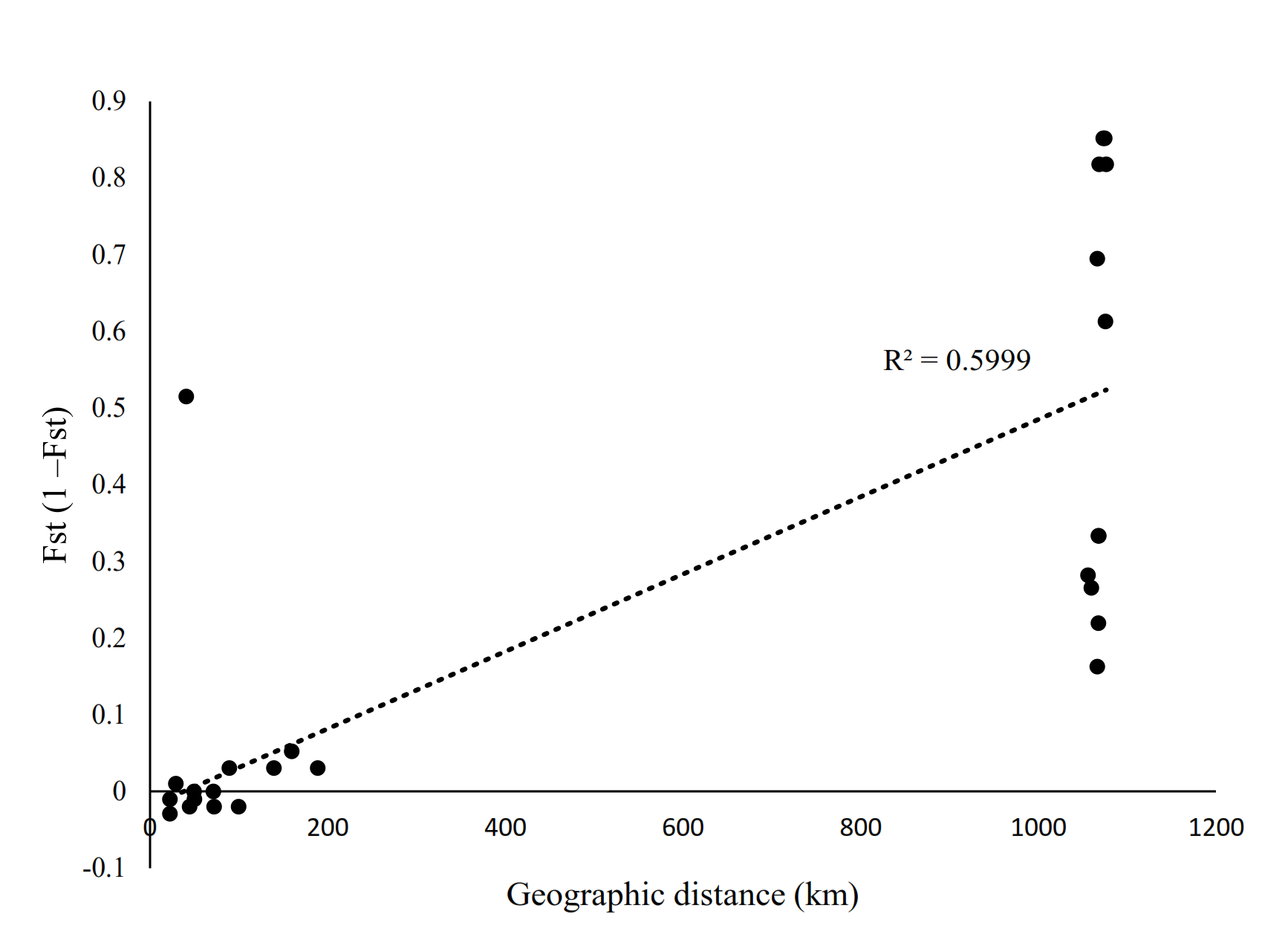

Supplement: S4 Fig — (TIF) [file pone.0285967.s004.tif]

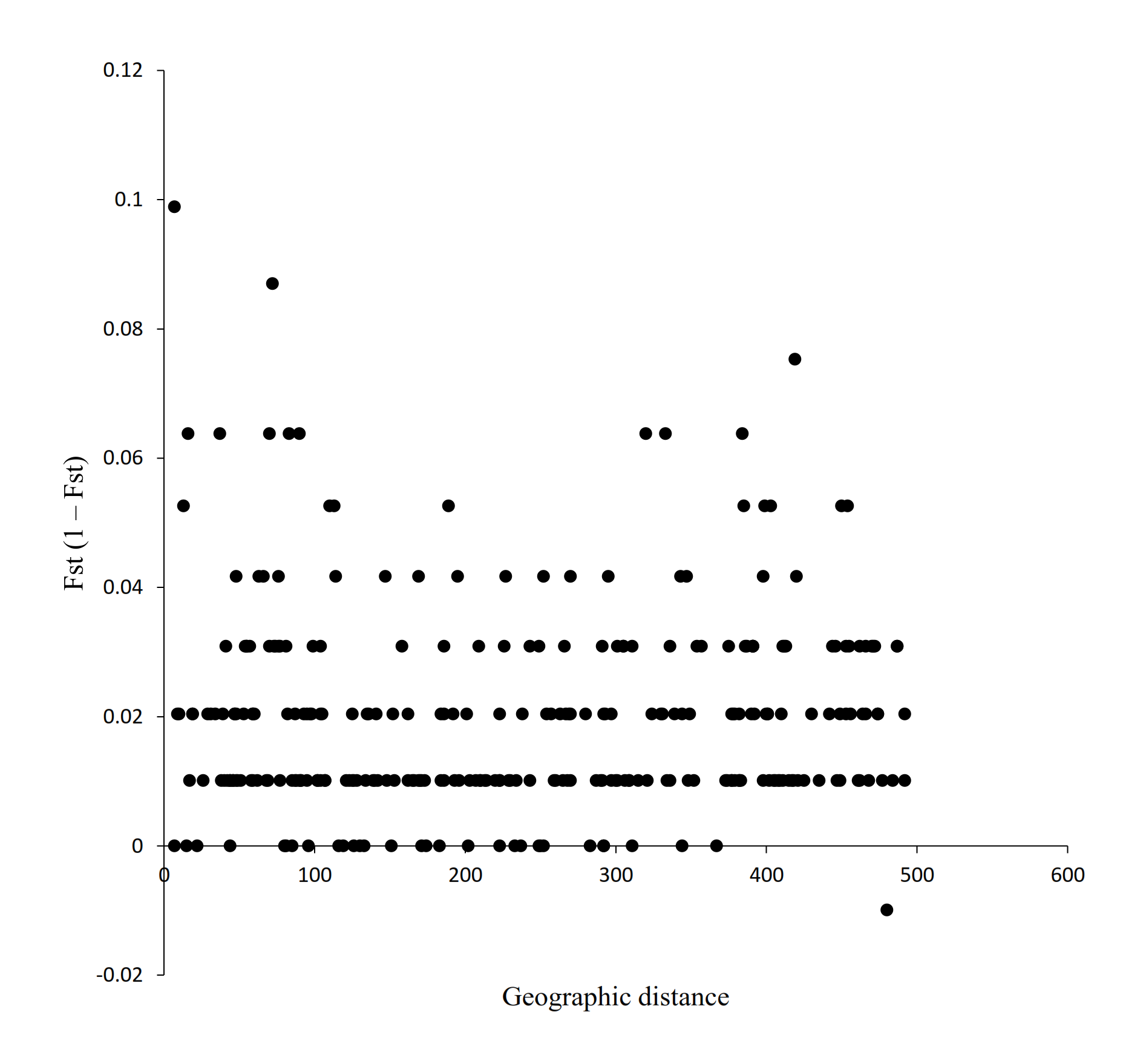

Supplement: S5 Fig — (TIF) [file pone.0285967.s005.tif]
