# Supplementary material for: Genetic diversity, distribution, and structure of Bemisia tabaci whitefly species in potential invasion and hybridization regions of East Africa
Source: PLoS One. 2023 May 25;18(5):e0285967. doi: 10.1371/journal.pone.0285967 (PMC10212157; doi:10.1371/journal.pone.0285967)
Supplement: S1 Table — Host plants and location of sampled adult B. tabaci and evidence of disease on cassava in A) Tanzania and B) Uganda. (ZIP) [file pone.0285967.s006.zip › Supp. Table 1A.docx]

A)

| Region | F/N | Date | Host plant | Host plant family | Host plant species | CV | MAP | Latitude | Longitude | WC | CMD | CBSD | SL |
| --- | --- | --- | --- | --- | --- | --- | --- | --- | --- | --- | --- | --- | --- |
| Arusha | F1 | 2/20/2016 | Tomato | Solanaceae | *Solanum lycopersicum* |  | 2 | S3°26.640' | E36°12.167' | 1 | - | - |  |
|  | F1 | 2/20/2016 | Eggplant | Solanaceae | *Solanum melongena* |  | 2 |  |  | 1 | - | - |  |
|  | F1 | 2/20/2016 | Sweetpotato | Convolvulaceae | *Ipomea batata* |  | 0.5 |  |  | 1 | - | - |  |
|  | F1 | 2/20/2016 | Fish poison | Fabaceae | *Tephrosia vogelii* |  |  |  |  | 1 | - | - |  |
|  | F1 | 2/20/2016 | Kale | Brassicaceae | *Brassica oleracea* |  | 0.75 |  |  | 1 | - | - |  |
|  | F1 | 2/20/2016 | Beans | Fabaceae | *Phaseolus vulgaris* |  | 1 |  |  | 1 | - | - |  |
|  | F2 | 2/20/2016 | Wireweed | Malvaceae | *Sida acuta* |  |  |  |  | 1 | - | - |  |
|  | F2 | 2/20/2016 | Moon flower | Solanceae | *Datura stramonium* |  |  |  |  | 1 | - | - |  |
| Manyara | F3 | 2/20/2016 | Cassava | Euphorbiaceae | *Manihot esculenta* | Wild cassava |  | S04°06.404' | E035°45.694' | 1 | 2 | 1 |  |
|  | F3 | 2/20/2016 | Pumpkin | Cucurbitaceae | *Cucurbita sp.* |  |  |  |  | 1 | - | - | NO |
|  | F3 | 2/20/2016 | Wireweed | Malvaceae | *Sida acuta* |  |  |  |  | 1 | - | - |  |
|  | F3 | 2/20/2016 | Eggplant | Solanaceae | *Solanum melongena* |  |  |  |  | 1 | - | - |  |
|  | F3 | 2/20/2016 | Fire plant | Euphorbiaceae | *Euphorbiaceae sp* |  |  |  |  | 1 | - | - |  |
|  | F4 | 2/20/2016 | Cassava | Euphorbiaceae | *Manihot esculenta* | Kigami | 7 | S04°06.765' | E035°40.296' | 1 | 2 | 1 |  |
|  | F4 | 2/20/2016 | Fish poison | Fabaceae | *Tephrosia vogelii* |  |  |  |  | 1 | - | - |  |
|  | F4 | 2/20/2016 | Groundnut | Fabaceae | *Arachis hypogaea* |  |  |  |  | 1 | - | - |  |
|  | F4 | 2/20/2016 | Bristly starbur | Asteraceae | *Acanthospermum hispidum* |  |  |  |  | 1 | - | - |  |
| Dodoma | F5 | 2/21/2016 | Cowpea | Fabaceae | *Vigna unguiculata* |  |  | S04°55.515' | E035°48.562' | 1 | - | - |  |
|  | F5 | 2/21/2016 | Sweetpotato | Convolvulaceae | *Ipomea batata* |  |  |  |  | 1 | - | - |  |
|  | F5 | 2/21/2016 | Pumpkin | Cucurbitaceae | *Cucurbita sp.* |  |  |  |  | 1 | - | - | YES |
|  | F6 | 2/21/2016 | Cassava | Euphorbiaceae | *Manihot esculenta* | Kaniki | 13 | S05°04.536' | E035°47.593' | 1 | 3 | 1 |  |
|  | F6 | 2/21/2016 | Groundnut | Fabaceae | *Arachis hypogaea* |  |  |  |  | 1 | - | - |  |
|  | F6 | 2/21/2016 | Dicotyledon weed | Unknown | Unknown |  |  |  |  | 1 | - | - |  |
|  | F6 | 2/20/2016 | Sesame | Pedaliaceae | *Sesamum indicum* |  |  |  |  | 1 | - | - |  |
|  | F7 | 2/21/2016 | Cassava | Euphorbiaceae | *Manihot esculenta* | Unknown | 5 | S05°50.783' | E035°45.450' | 1 | 3 | 1 |  |
|  | F7 | 2/21/2016 | Sesame | Pedaliaceae | *Sesamum indicum* |  |  |  |  | 10 | - | - |  |
|  | F7 | 2/21/2016 | Spider flower | Cleomaceae | *Cleome viscosa* |  |  |  |  | 1 | - | - |  |
|  | F7 | 2/20/2017 | Cucumber | Cucurbitaceae | *Cucumis sativus* |  |  |  |  | 1 | - | - |  |
|  | F7 | 2/21/2016 | Sweetpotato | Convolvulaceae | *Ipomea batata* |  |  |  |  | 1 | - | - |  |
|  | F7 | 2/21/2016 | Pink morning glory | Convolvulaceae | *Ipomea carnea* |  |  |  |  | 1 | - | - |  |
|  | F8 | 2/21/2016 | Watermelon | Convolvulaceae | *Citrullus lanatus* |  |  | S06°07.461' | E035°48.471' | 1 | - | - |  |
|  | F8 | 2/21/2016 | Azanza | Malvaceae | *Thespesia garckeana* |  |  |  |  | 1 | - | - |  |
|  | F8 | 2/21/2016 | Cassava | Euphorbiaceae | *Manihot esculenta* | Unknown | 2 |  |  | 1 | 2 | 1 |  |
|  | F8 | 2/21/2016 | Bristly starbur | Asteraceae | *Acanthospermum hispidum* |  |  |  |  | 1 | - | - |  |
|  | F8 | 2/21/2016 | Cowpea | Fabaceae | *Vigna unguiculata* |  |  |  |  | 1 | - | - |  |
|  | F8 | 2/21/2016 | Groundnut | Fabaceae | *Arachis hypogaea* |  |  |  |  | 1 | - | - |  |
|  | F8 | 2/21/2016 | Tomato | Solanaceae | *Solanum lycopersicum* |  |  |  |  | NA | - | - |  |
|  | F8 | 2/21/2016 | Okra | Malvaceae | *Abelmoschus esculentus* |  |  |  |  | 1 | - | - |  |
|  | F8 | 2/21/2016 | Morning glory | Convolvulaceae | *Ipomea sp* |  |  |  |  | 1 | - | - |  |
|  | F8 | 2/21/2016 | Green gram | Fabaceae | *Vigna radiata* |  |  |  |  | 1 | - | - |  |
|  | F9 | 2/22/2016 | Groundnut | Fabaceae | *Arachis hypogaea* |  |  | S06°07.497' | E036°12.121' | 10 | - | - |  |
|  | F9 | 2/22/2016 | Cowpea | Fabaceae | *Vigna unguiculata* |  |  |  |  | 10 | - | - |  |
|  | F9 | 2/22/2016 | Sunflower | Asteraceae | *Helianthus annuus* |  |  |  |  | 10 | - | - |  |
|  | F9 | 2/22/2016 | Morning glory | Convolvulaceae | *Ipomea sp* |  |  |  |  | 10 | - | - |  |
|  | F9 | 2/22/2016 | Wireweed | Malvaceae | *Sida acuta* |  |  |  |  | 10 | - | - |  |
|  | F10 | 2/22/2016 | Cassava | Euphorbiaceae | *Manihot esculenta* | unknown | 11 | S06°02.759' | E036°35.372' | 1 | 1 | 1 |  |
|  | F10 | 2/22/2016 | Sunflower | Asteraceae | *Helianthus annuus* |  |  |  |  | 1 | - | - |  |
|  | F10 | 2/22/2016 | Watermelon | Convolvulaceae | *Citrullus lanatus* |  |  |  |  | 1 | - | - |  |
|  | F10 | 2/22/2016 | Okra | Malvaceae | *Abelmoschus esculentus* |  |  |  |  | 1 | - | - |  |
|  | F10 | 2/22/2016 | Cowpea | Fabaceae | *Vigna unguiculata* |  |  |  |  | 1 | - | - |  |
|  | F10 | 2/22/2016 | Pumpkin | Cucurbitaceae | *Cucurbita sp.* |  |  |  |  | 1 | - | - | NO |
|  | F10 | 2/22/2016 | Dockeweed | Polygonaceae | *Oxygonum sp.* |  |  |  |  | 1 | - | - |  |
| Morogoro | F11 | 2/22/2016 | Spider flower | Cleomaceae | *Cleome viscosa* |  |  | S06°08.243' | E036°56.220' | 1 | - | - |  |
|  | F11 | 2/22/2016 | Cassava | Euphorbiaceae | *Manihot esculenta* | Kaniki | 11 |  |  | 1 | 2 | 1 |  |
|  | F11 | 2/22/2016 | Rattlepods | Fabaceae | *Crotalaria sp.* |  |  |  |  | 1 | - | - |  |
|  | F11 | 2/22/2016 | Pumpkin | Cucurbitaceae | *Cucurbita sp.* |  |  |  |  | 1 | - | - | YES |
|  | F11 | 2/22/2016 | Moon flower | Solanaceae | *Datura stramonium* |  |  |  |  | 1 | - | - |  |
|  | F11 | 2/22/2016 | Fire plant | Euphorbiaceae | *Euphorbiaceae sp* |  |  |  |  | 10 | - | - |  |
|  | F11 | 2/22/2016 | Cowpea | Fabaceae | *Vigna unguiculata* |  |  |  |  | 1 | - | - |  |
|  | F11 | 2/22/2016 | Azanza | Malvaceae | *Thespesia garckeana* |  |  |  |  | 1 | - | - |  |
|  | F11 | 2/22/2016 | Sweetpotato | Convolvulaceae | *Ipomea batata* |  |  |  |  | 1 | - | - |  |
|  | F12 | 2/22/2016 | Spider flower | Cleomaceae | *Cleome viscosa* |  |  | S06°22.700' | E37°19.733' | 10 | - | - |  |
|  | F12 | 2/22/2016 | Cassava | Euphorbiaceae | *Manihot esculenta* | Unknown | 2 |  |  | 100 | 2 | 2 |  |
|  | F12 | 2/22/2016 | Erect boerhavia | Nyctaginaceae | *Boerhavia erecta* |  |  |  |  | 1 | - | - |  |
|  | F12 | 2/22/2016 | Wireweed | Malvaceae | *Sida acuta* |  |  |  |  | 10 | - | - |  |
|  | F13 | 2/22/2016 | Cowpea | Fabaceae | *Vigna unguiculata* |  | 2 | S06°40.550' | E37°37.517' | 1 | - | - |  |
|  | F13 | 2/22/2016 | Cassava | Euphorbiaceae | *Manihot esculenta* | Shinatenga | 3 |  |  | 10 | 1 | 1 |  |
|  | F13 | 2/22/2016 | Pumpkin | Cucurbitaceae | *Cucurbita sp.* |  | 1.5 |  |  | 100 | - | - | YES |
|  | F13 | 2/22/2016 | Sunflower | Asteraceae | *Helianthus annuus* |  |  |  |  | 1 | - | - |  |
|  | F13 | 2/22/2016 | Okra | Malvaceae | *Abelmoschus esculentus* |  |  |  |  | 10 | - | - |  |
|  | F13 | 2/22/2016 | Erect boerhavia | Nyctaginaceae | *Boerhavia erecta* |  |  |  |  | 10 | - | - |  |
|  | F14 | 2/23/2016 | Woody vines | Fabaceae | *Cissus sp.* |  |  | S06°40.670' | E37°58.846' | 10 | - | - |  |
|  | F14 | 2/23/2016 | Erect boerhavia | Nyctaginaceae | *Boerhavia erecta* |  |  |  |  | 10 | - | - |  |
|  | F14 | 2/23/2016 | Cassava | Euphorbiaceae | *Manihot esculenta* | Kikombe | 3 |  |  | 100 | 3 | 2 |  |
|  | F14 | 2/23/2016 | Cowpea | Fabaceae | *Vigna unguiculata* |  | 3 |  |  | 500 | - | - |  |
|  | F14 | 2/23/2016 | Wireweed | Malvaceae | *Sida acuta* |  |  |  |  | 100 | - | - |  |
|  | F14 | 2/23/2016 | Fish poison | Fabaceae | *Tephrosia vogelii* |  |  |  |  | 100 | - | - |  |
|  | F14 | 2/23/2016 | Bristly starbur | Asteraceae | *Acanthospermum hispidum* |  |  |  |  | 10 | - | - |  |
|  | F14 | 2/23/2016 | Okra | Malvaceae | *Abelmoschus esculentus* |  |  |  |  | 1 | - | - |  |
|  | F14 | 2/23/2016 | Pumpkin | Cucurbitaceae | *Cucurbita sp.* |  |  |  |  | 10 | - | - | YES |
| Pwani | F15 | 2/23/2016 | Okra | Malvaceae | *Abelmoschus esculentus* |  |  | S06°38.470' | E38°23.393' | 1 | - | - |  |
|  | F15 | 2/23/2016 | Cassava | Euphorbiaceae | *Manihot esculenta* | Ndope | 9 |  |  | 100 | 1 | 1 |  |
|  | F15 | 2/23/2016 | Pumpkin | Cucurbitaceae | *Cucurbita sp.* |  |  |  |  | 10 | - | - | NO |
|  | F15 | 2/23/2016 | Wireweed | Malvaceae | *Sida acuta* |  |  |  |  | 10 | - | - |  |
|  | F15 | 2/23/2016 | Bush weed |  | *Unknown* |  |  |  |  | 10 | - | - |  |
|  | F15 | 2/23/2016 | Cowpea | Fabaceae | *Vigna unguiculata* |  |  |  |  | 10 | - | - |  |
| DSM | F16 | 2/23/2016 | Cassava | Euphorbiaceae | *Manihot esculenta* | Unknown | 4 | S06°43.502' | E39°06.782' | 100 | 4 | 3 |  |
|  | F16 | 2/23/2016 | Watermelon | Convolvulaceae | *Citrullus lanatus* |  |  |  |  | 10 | - | - |  |
|  | F17 | 2/23/2016 | Cassava | Euphorbiaceae | *Manihot esculenta* | Ndope | 9 | S06°47.333' | E39°06.784' | 10 | 3 | 1 |  |
|  | F17 | 2/23/2016 | Spider flower | Cleomaceae | *Cleome viscosa* |  |  |  |  | 1 | - | - |  |
|  | F17 | 2/23/2016 | Pumpkin | Cucurbitaceae | *Cucurbita sp.* |  |  |  |  | 10 | - | - | YES |
|  | F17 | 2/23/2016 | Erect boerhavia | Nyctaginaceae | *Boerhavia erecta* |  |  |  |  | 1 | - | - |  |
|  | F17 | 2/23/2016 | Fire plant | Euphorbiaceae | *Euphorbiaceae sp* |  |  |  |  | 10 | - | - |  |
|  | F17 | 2/23/2016 | Cabbage | Brassicaceae | *Brassica oleracea* |  |  |  |  | 10 | - | - |  |
| Pwani | F18 | 2/24/2016 | Cassava | Euphorbiaceae | *Manihot esculenta* | Kiroba | 11 | S06°35.558' | E39°04.878' | 100 | 3 | 2 |  |
|  | F18 | 2/24/2016 | Pumpkin | Cucurbitaceae | *Cucurbita sp.* |  |  |  |  | 1 | - | - | YES |
|  | F18 | 2/24/2016 | Okra | Malvaceae | *Abelmoschus esculentus* |  |  |  |  | 1 | - | - |  |
|  | F18 | 2/24/2016 | Bush weed |  | *Unknown* |  |  |  |  | 1 | - | - |  |
|  | F18 | 2/24/2016 | Cassias | Fabaceae | *Cassia sp.* |  |  |  |  | 1 | - | - |  |
|  | F19 | 2/24/2016 | Cassava | Euphorbiaceae | *Manihot esculenta* | Mfawima | 4 | S06°27.821' | E38°53.364' | 1 | 2 | 1 |  |
|  | F19 | 2/24/2016 | Cowpea | Fabaceae | *Vigna unguiculata* |  |  |  |  | 1 | - | - |  |
|  | F19 | 2/24/2016 | Fish poison | Fabaceae | *Tephrosia vogelii* |  |  |  |  | 10 | - | - |  |
|  | F19 | 2/24/2016 | Pumpkin | Cucurbitaceae | *Cucurbita sp.* |  |  |  |  | 1 | - | - | NO |
|  | F19 | 2/24/2016 | Watermelon | Convolvulaceae | *Citrullus lanatus* |  |  |  |  | 10 | - | - |  |
|  | F20 | 2/24/2016 | Cassava | Euphorbiaceae | *Manihot esculenta* | Mfaransa | 3 | S06°22.744' | E38°35.741' | 10 | 3 | 1 |  |
|  | F20 | 2/24/2016 | Pignut | Lamiaceae | *Carya glabra* |  |  |  |  | 100 | - | - |  |
|  | F21 | 2/24/2016 | Cassava | Euphorbiaceae | *Manihot esculenta* | Mzuri kuonja | |  |  | 500 | 4 | 4 |  |
|  | F21 | 2/24/2016 | Bush weed |  | Unknown |  |  |  |  | 100 | - | - |  |
|  | F21 | 2/24/2016 | Green gram | Fabaceae | *Vigna radiata* |  |  |  |  | 10 | - | - |  |
|  | F21 | 2/24/2016 | Morning glory | Convolvulaceae | *Ipomea sp* |  |  |  |  | 100 | - | - |  |
| Zanzibar | F22 | 2/25/2016 | Cassava | Euphorbiaceae | *Manihot esculenta* | Mwali | 5 | S6°9.955' | E39°12.158' | 10 | 2 | 1 |  |
|  | F23 | 2/25/2016 | Fish poison | Fabaceae | *Tephrosia vogelii* |  |  |  |  | 1 | - | - |  |
|  | F23 | 2/25/2016 | Okra | Malvaceae | *Abelmoschus esculentus* |  |  |  |  | 1 | - | - |  |
|  | F23 | 2/25/2016 | Sweetpotato | Convolvulaceae | *Ipomea batata* |  |  |  |  | 1 | - | - |  |
|  | F23 | 2/25/2016 | Cassava | Euphorbiaceae | *Manihot esculenta* | Muafaka | 3 |  |  | 100 | 3 | 3 |  |
|  | F23 | 2/25/2016 | Watermelon | Convolvulaceae | *Citrullus lanatus* |  |  |  |  | 1 | - | - |  |
|  | F23 | 2/25/2016 | Zucchini | Cucurbitaceae | *Cucurbita pepo* |  |  |  |  | 1 | - | - |  |
|  | F24 | 2/25/2016 | Cassava | Euphorbiaceae | *Manihot esculenta* | Mwali | 4 | S06°16.117' | E039°15.633' | 10 | - | - |  |
|  | F24 | 2/25/2016 | Wireweed | Malvaceae | *Sida acuta* |  |  |  |  | 1 | - | - |  |
|  | F24 | 2/25/2016 | Okra | Malvaceae | *Abelmoschus esculentus* |  | 3 |  |  | 1 | 2 | 1 |  |
|  | F24 | 2/25/2016 | Sweet pepper | Solanaceae | *Capsicum annuum* |  | 0.5 |  |  | 1 | - | - |  |
|  | F24 | 2/25/2016 | Tomato | Solanaceae | *Solanum lycopersicum* |  | 3 |  |  | 1 | - | - |  |
|  | F24 | 2/25/2016 | Eggplant | Solanaceae | *Solanum melongena* |  | 2.5 |  |  | 10 | - | - |  |
|  | F25 | 2/26/2016 | Fire plant | Euphorbiaceae | *Euphorbiaceae sp.* |  |  | S06°14.339' | E039°19.743' | 1 | - | - |  |
|  | F25 | 2/26/2016 | Watermelon | Cucurbitaceae | *Citrullus lanatus* |  |  |  |  | 1 | - | - |  |
|  | F25 | 2/26/2016 | Cassava | Euphorbiaceae | *Manihot esculenta* | Unknown | 6 |  |  | 0 | 1 | 1 |  |
|  | F25 | 2/26/2016 | Tomato | Solanaceae | *Solanum lycopersicum* |  |  |  |  | 1 | - | - |  |
|  | F26 | 2/26/2016 | Cassava | Euphorbiaceae | *Manihot esculenta* | Kizimbani | 7 | S06°6.186' | E039°13.474' | 10 | 1 | 1 |  |
|  | F26 | 2/26/2016 | Annual poinsetia | Euphorbiaceae | *Euphorbia heterophylla* |  |  |  |  | 10 | - | - |  |
|  | F26 | 2/26/2016 | Cucumber | Cucurbitaceae | *Cucumis sativus* |  |  |  |  | 1 | - | - |  |
|  | F27 | 2/27/2016 | Cassava | Euphorbiaceae | *Manihot esculenta* | Mwali/Joya | 4 |  |  | 100 | 1 | 2 |  |
|  | F27 | 2/27/2016 | Caesarweed | Malvaceae | *Urena lobate* |  |  |  |  | 100 | - | - |  |

FN: field number; CV: cassava variety name; MAP: age of cassava (month after planting); WC: whitefly count (>100 regarded as superabundant,); CMD and CBSD: presence of disease symptoms in field grown cassava scored in 1 – 5 scale; SL: silver leafing in field grown pumpkin, DSM: Dar es Salaam; ``–´´ data not available and “^1”^ data from cassava are from Ally et al. [44].
