# Supplementary material for: Genetic diversity, distribution, and structure of Bemisia tabaci whitefly species in potential invasion and hybridization regions of East Africa
Source: PLoS One. 2023 May 25;18(5):e0285967. doi: 10.1371/journal.pone.0285967 (PMC10212157; doi:10.1371/journal.pone.0285967)
Supplement: S1 Table — Host plants and location of sampled adult B. tabaci and evidence of disease on cassava in A) Tanzania and B) Uganda. (ZIP) [file pone.0285967.s006.zip › Supp. Table 1B.docx]

B)

| District | F/N | Date | Host plant common name | Host plant family | Host plant species | CV | MAP | Latitude | Longitude | WC | CMD | CBSD |
| --- | --- | --- | --- | --- | --- | --- | --- | --- | --- | --- | --- | --- |
| Mityana | F1 | 2/8/2017 | Cassava | Euphorbiaceae | *Manihot esculenta^1^* | Gomboka | 7 | N00.43564 | E032.04041 | 100 | 3 | 1 |
|  | F1 | 2/8/2017 | Sweet potato | Convolvulaceae | *Ipomea batata* |  |  | N00.43564 | E032.04041 | 1 | - | - |
|  | F1 | 2/8/2017 | Tickberry | Verbenaceae | *Lantana camara* |  |  | N00.43564 | E032.04041 | 1 | - | - |
| Mpiji | F2 | 2/8/2017 | Cassava | Euphorbiaceae | *Manihot esculenta^1^* | Akena | 6 | N00.00979 | E032.00677 | 100 | 1 | 3 |
|  | F2 | 2/8/2017 | Pumpkin | Cucurbitaceae | *Cucurbita sp.* |  |  | N00.00979 | E032.00677 | 1 | - | - |
|  | F2 | 2/8/2017 | Eggplant | Solanaceae | *Solanum melongena* |  |  | N00.00979 | E032.00677 | 1 | - | - |
|  | F2 | 2/8/2017 | Sweet potato | Convolvulaceae | *Ipomea batata* |  |  | N00.00979 | E032.00677 | 1 | - | - |
| Wakiso | F3 | 2/9/2017 | Cassava | Euphorbiaceae | *Manihot esculenta^1^* | NASE 3 | 2.5 | N00.51831 | E032.63553 | 1 | 1 | 1 |
|  | F4 | 2/9/2017 | Lion's ear | Lamiaceae | *Leonotis leonurus* |  |  | N00.52020 | E032.63834 | 1 | - | - |
| Kalungu | F5 | 2/10/2017 | Cassava | Euphorbiaceae | *Manihot esculenta^1^* | TME 14 | 4 | S00.16989 | E031.83412 | 1 | 4 | 3 |
|  | F5 | 2/10/2017 | Sweet potato | Convolvulaceae | *Ipomea batata* |  |  | S00.16989 | E031.83412 | 1 | - | - |
| Masaka | F6 | 2/10/2017 | Cassava | Euphorbiaceae | *Manihot esculenta^1^* | Unknown | 6 | S00.33294 | E031.70984 | 10 | 3 | 3 |
|  | F6 | 2/10/2017 | Pumpkin | Cucurbitaceae | *Cucurbita sp.* |  |  | S00.33294 | E031.70984 | 1 | - | - |
|  | F6 | 2/10/2017 | Sweet potato | Convolvulaceae | *Ipomea batata* |  |  | S00.33294 | E031.70984 | 1 | - | - |
| Rakai | F7 | 2/10/2017 | Cassava | Euphorbiaceae | *Manihot esculenta^1^* | Unknown | 6 | S00.52627 | E031.64813 | 100 | 2 | 2 |
|  | F7 | 2/10/2017 | Sweet potato | Convolvulaceae | *Ipomea batata* |  |  | S00.52627 | E031.64813 | 1 | - | - |
|  | F7 | 2/10/2017 | Beans | Fabaceae | *Phaseolus vulgaris* |  |  | S00.52627 | E031.64813 | 1 | - | - |
| Rakai | F8 | 2/10/2017 | Cassava | Euphorbiaceae | *Manihot esculenta^1^* | TME 204 | 3 | S00.66515 | E031.53927 | 10 | 2 | 2 |
|  | F8 | 2/10/2017 | Black-jack | Asteraceae | *Bidens Pilosa* |  |  | S00.66515 | E031.53927 | 1 | - | - |
| Rakai | F9 | 2/11/2017 | Cassava | Euphorbiaceae | *Manihot esculenta^1^* | TME 14 | 4 | S00.69034 | E031.43948 | 10 | 1 | 1 |
|  | F9 | 2/11/2017 | Sweet potato | Convolvulaceae | *Ipomea batata* |  |  | S00.69034 | E031.43948 | 1 | - | - |
| Rakai | F10 | 2/11/2017 | Cassava | Euphorbiaceae | *Manihot esculenta^1^* | Kalandila | 7 | S00.89538 | E031.44637 | 100 | 3 | 1 |
|  | F10 | 2/11/2017 | Wandering jew | Commelinaceae | *Commelina benghalensis* |  |  | S00.89538 | E031.44637 | 1 | - | - |
| Rakai | F11 | 2/12/2017 | Cassava | Euphorbiaceae | *Manihot esculenta^1^* | TME 14 | 6 | S00.98063 | E031.41873 | 500 | 4 | 2 |
|  | F11 | 2/12/2017 | Black-jack | Asteraceae | *Bidens Pilosa* |  |  | S00.98063 | E031.41873 | 1 | - | - |
| Kalungu | F12 | 2/12/2017 | Cassava | Euphorbiaceae | *Manihot esculenta^1^* | TME 14 | 3 | S00.12179 | E031.75773 | 500 | 1 | 1 |
|  | F12 | 2/12/2017 | Pokeweeds | Phytolacaceae | *Phytolacca decandra* |  |  | S00.12179 | E031.75773 | 1 | - | - |
| Gomba | F13 | 2/12/2017 | Cassava | Euphorbiaceae | *Manihot esculenta^1^* | NASE 3 | 5 | N00.17379 | E031.92822 | 100 | 1 | 1 |
| Wakiso | F14 | 2/13/2017 | Sweet potato | Convolvulaceae | *Ipomea batata* |  |  | N00.52556 | E032.62680 | 1 | - | - |
|  | F14 | 2/13/2017 | Cassava | Euphorbiaceae | *Manihot esculenta^1^* | NAROCAS 2 | 8 | N00.52556 | E032.62680 | 100 | 3 | 1 |
|  | F14 | 2/13/2017 | Indian Mustard | Brassicaceae | *Brassica juncea* |  |  | N00.52556 | E032.62680 | 1 | - | - |

FN: field number; CV: cassava variety name; MAP: age of cassava (month after planting); WC: whitefly count (>100 regarded as superabundant,); CMD and CBSD: presence of disease symptoms in field grown cassava scored in 1 – 5 scale; SL: silver leafing in field grown pumpkin, DSM: Dar es Salaam; ``–´´ data not available and “^1”^ data from cassava are from Ally et al. [44].
