# Supplementary material for: Genetic diversity, distribution, and structure of Bemisia tabaci whitefly species in potential invasion and hybridization regions of East Africa
Source: PLoS One. 2023 May 25;18(5):e0285967. doi: 10.1371/journal.pone.0285967 (PMC10212157; doi:10.1371/journal.pone.0285967)
Supplement: S2 Table — (DOCX) [file pone.0285967.s007.docx]

| **LN** | **Reference** | **Primer sequence** | **Motif** | **FL** | **%MS** | **Range** |
| --- | --- | --- | --- | --- | --- | --- |
| MS145 | [44] | F: CCTACCCATGAGAGCGGTAA | (AC)9 | PET | 4.2 | 124-234 |
|  |  | R: TCAACAAACGCGTTCTTCAC |  |  |  |  |
| P59 | [81] | F: CGGCGTTTCTCGTTTTCTT | (T)44(G)18 | 6-FAM | 2.3 | 148-220 |
|  |  | R: TTTGCCAACTGAAGCACATCAATCA |  |  |  |  |
| P7 | [81] | F: AGGGTGTCAGGTCAGGTAGC | 8(GT) | VIC | 2 | 117-287 |
|  |  | R: TTTGCGTAATAGAAAA |  |  |  |  |
| WF2H06 | [46] | F: TATTCGCCAATCGATTCCTT | (TTTG)11 | NED | 4.4 | 102-214 |
|  |  | R: CGGCGGAAATTTCGATAAA |  |  |  |  |
| P62 | [81] | F: CTTCCTTAGCACGGCAGAAT | (GT)8 | 6-FAM | 2.2 | 126-288 |
|  |  | R: TTTGGCGCAATTTTTAGCGTCTGT |  |  |  |  |
| WF1G03 | [46] | F: CTCCAAAATGGGACTTGAAC | (GTTT)8 | PET | 1.2 | 102-292 |
|  |  | R: GTAGAAGCCACACATACTAGCAC |  |  |  |  |
| WF1D04 | [46] | F: GTTGTTAGGTTACAGGGTTTGTC | (CAAA)16 | VIC | 1.8 | 100-172 |
|  |  | R: GTCTTTACTTCTTTTCCTCCG |  |  |  |  |
| P5 | [81] | F: ATTAGCCTTGCTTGGGTCCT | (GT)8 | NED | 4.8 | 100-288 |
|  |  | R: TTTGCAAAAACAAAAGCATGTGTCAAA |  |  |  |  |
| CIRSSA2 | [37] | F: ACAATGCATGTTGATTGTGAA | (AG)6 | VIC | 0.2 | 100-126 |
|  |  | R: TGAAAATGTCTACGGCCAGA |  |  |  |  |
| CIRSSA6 | [37] | F: CATATCGGTCATTATCCGCA | (TC)6 | VIC | 0.2 | 117-173 |
|  |  | R: CATCAGGCTGGAAAGACGAG |  |  |  |  |
| CIRSSA7 | [37] | F: TGGCGATCCTCTTCTTGTTT | (TC)5 | PET | 0.2 | 122-152 |
|  |  | R: AAGAAGCAGCAGTTCATCCG |  |  |  |  |
| CIRSSA13 | [37] | F: AGTGCTGAAGGTCCACCGTA | (CT)6 | NED | 12.8 | 145-291 |
|  |  | R: GGGATTTCCAGGGGTTAAGA |  |  |  |  |
| CIRSSA41 | [37] | F: TGGGTGCATGGTTCTTACAG | (CT)6 | 6-FAM | 25 | 112-184 |
|  |  | R: TATCCGGTCGACAAACACAA |  |  |  |  |

Locus name (LN), source reference, primer sequence, microsatellite repeat motif, fluorochromes used for labelling primers (FL), percentage of missing data in the whole dataset (%MS), allele size range (Range, bp).
