# Supplementary material for: Genetic diversity, distribution, and structure of Bemisia tabaci whitefly species in potential invasion and hybridization regions of East Africa
Source: PLoS One. 2023 May 25;18(5):e0285967. doi: 10.1371/journal.pone.0285967 (PMC10212157; doi:10.1371/journal.pone.0285967)
Supplement: S3 Table — (DOCX) [file pone.0285967.s008.docx]

|  | Field observed | Field observed | Number of haplotypes per country | | Accession |
| --- | --- | --- | --- | --- | --- |
| Haplotype name | Tanzania | Uganda | Tanzania | Uganda | Number |
| P10G3_UG | F3, F4, F8 | F1, F2, F3, F5, | 7 | 146 | MN709409 |
|  |  | F6, F7, F8, F9, |  |  |  |
|  |  | F10, F11, F12, |  |  |  |
|  |  | F13, F14 |  |  |  |
| P5B11_TZ | F4, F8 |  | 4 |  | MN709411 |
| P5D9_TZ | F8 |  | 1 |  | MN709412 |
| P4A3_TZ | F6, F7, F8, | F1, F2, F3, F5, | 31 | 130 | MN709403 |
|  |  | F6, F7, F8, F9, |  |  |  |
|  |  | F10, F11, F12, |  |  |  |
|  |  | F13, F14 |  |  |  |
| P6F2_TZ | F4, F6, F8 |  | 19 |  | MN709404 |
| P9B1_UG |  | F1, F2, F5 |  | 4 | MK360167 |
| P4H3_TZ | F6 |  | 1 |  | MN709405 |
| P5A1_UG |  | F5 |  | 1 | MN709407 |
| P4H11_UG |  | F5 |  | 1 | MN709408 |
| P6H2_TZ | F8 |  | 1 |  | MN709406 |
| P22G3_TZ | F16, F17, F18, F19, |  | 55 |  | MN709401 |
| P17C8_TZ | F20, F21, F26, F27 |  | 3 |  | MN709402 |
| P26B10_TZ | F16, F17, F18, F27 |  | 1 |  | MN709400 |
| P5H7_UG |  | F6, F7, F9, F1, F14 |  | 11 | MN709431 |
| P22E1_TZ | F25, F15, F19 |  | 8 |  | MN709426 |
| P22B1_TZ | F16, F17, F25 |  | 5 |  | MN709428 |
| P3E11_UG |  | F2, F4 |  | 3 | MN709429 |
| P6F1_UG |  | F7 |  | 2 | MN709432 |
| P17F10_TZ | F18 |  | 1 |  | MN709427 |
| P16B6_TZ | F16 |  | 1 |  | MN709430 |
| P17H10_TZ | F18 |  | 1 |  | MN709423 |
| P3C12_UG |  |  |  | 1 | MN709424 |
| P22C12_TZ | F27 |  | 1 |  | MN709420 |
| P17E9_TZ | F18 |  | 1 |  | MN709421 |
| P22H12_TZ | F27 |  | 1 |  | MN709422 |
| P22D11_TZ | F27 |  | 4 |  | MN709419 |
| P17F12_TZ | F18 |  | 1 |  | MN709425 |
| P4B7_UG |  | F1, F4 |  | 3 | MN709413 |
| P7C5_UG |  | F10 |  | 1 | MN709441 |
| P3G5_UG |  | F1, F2 |  | 3 | MN709439 |
| P4F7_UG |  | F4 |  | 1 | MN709436 |
| P3B7_UG |  | F1 |  | 1 | MN709435 |
| P4H6_UG |  | F4 |  | 1 | MN709437 |
| P3E3_UG |  | F1 |  | 1 | MN709438 |
| P3F7_UG |  | F1 |  | 1 | MN709440 |
| P3E5-_UG |  | F1, F2, F5, F6, F7, |  | 17 | MN709415 |
|  |  | F8, F9, F11, F14 |  |  |  |
| P8C9_UG |  | F14 |  | 3 | MN709416 |
| P3A4_UG |  | F1 |  | 1 | MN709417 |
| P3D2_TZ | F1, F2, F3, F4, |  | 330 |  | MN709444 |
|  | F5, F6, F7, F8 |  |  |  |  |
|  | F14, F17, F18, |  |  |  |  |
|  | F24 |  |  |  |  |
|  |  |  |  |  |  |
|  |  |  |  |  |  |
|  |  |  |  |  |  |
| P2B3_TZ | F1, F2, F3, F4, |  | 155 |  | MN709472 |
|  | F5, F6, F7, F8, |  |  |  |  |
|  | F9, F10, F14, |  |  |  |  |
|  | F17, F20, F24, |  |  |  |  |
|  | F26, F24, F25 |  |  |  |  |
|  |  |  |  |  |  |
|  |  |  |  |  |  |
| P22H1_TZ | F1, F2, F4, F5 |  | 31 |  | MN709483 |
|  | F7, F8, F9, |  |  |  |  |
|  | F14, F17, F18, |  |  |  |  |
|  |  |  |  |  |  |
| P1H4_TZ | F1, F2, F7, F9, F17 |  | 5 |  | MN709457 |
| P1H7_TZ | F1, F3, F8 |  | 3 |  | MN709467 |
| P6D12_TZ | F1, F9, F14 |  | 3 |  | MN709484 |
| P7A2_TZ | F9, F14 |  | 2 |  | MN709470 |
| P6B9_TZ | F8 | F12 | 1 | 1 | MN709481 |
| P5F1_TZ | F5, F6 |  | 2 |  | MN709489 |
| P3B2_TZ | F3 |  | 1 |  | MN709449 |
| P17A4_TZ | F17 |  | 1 |  | MN709455 |
| P5H11_TZ | F9 |  | 1 |  | MN709456 |
| P4C7_TZ | F6 |  | 1 |  | MN709461 |
| P1D8_TZ | F1 |  | 1 |  | MN709458 |
| P7D5_TZ | F9 |  | 1 |  | MN709459 |
| P6F5_TZ | F8 |  | 1 |  | MN709453 |
| P17A2_TZ | F17 |  | 1 |  | MN709445 |
| P5C12_TZ | F8 |  | 1 |  | MN709452 |
| P3G10_TZ | F5 |  | 1 |  | MN709451 |
| P6E12_TZ | F9 |  | 1 |  | MN709460 |
| P3A2_TZ | F3 |  | 1 |  | MN709448 |
| P6E6_TZ | F8 |  | 1 |  | MN709447 |
| P7E11_TZ | F9 |  | 1 |  | MN709450 |
| P7D6_TZ | F9 |  | 1 |  | MN709442 |
| P2D12_TZ | F3 |  | 1 |  | MN709443 |
| P1D5_TZ | F1 |  | 1 |  | MN709446 |
| P3E9_TZ | F4 |  | 1 |  | MN709469 |
| P13H11_TZ | F14 |  | 1 |  | MN709454 |
| P7F4_TZ | F17 |  | 1 |  | MN709466 |
| P1D2_TZ | F1 |  | 1 |  | MN709465 |
| P3C1_TZ | F3 |  | 1 |  | MN709464 |
| P17F6_TZ | F17 |  | 1 |  | MN709463 |
| P21D11_TZ | F25 |  | 1 |  | MN709462 |
| P7C1_TZ | F9 |  | 1 |  | MN709468 |
| P7F10_TZ | F9 |  | 1 |  | MN709471 |
| P7A6_TZ | F9 |  | 1 |  | MN709475 |
| P14H11_TZ | F14 |  | 1 |  | MN709478 |
| P14G11_TZ | F14 |  | 1 |  | MN709476 |
| P6C12_TZ | F9 |  | 1 |  | MN709474 |
| P3F8_TZ | F4 |  | 1 |  | MN709473 |
| P6C8_TZ | F8 |  | 1 |  | MN709477 |
| P2D10_TZ | F3 |  | 1 |  | MN709479 |
| P5A1_TZ | F7 |  | 1 |  | MN709480 |
| P6D6_TZ | F8 |  | 1 |  | MN709487 |
| P7D1_TZ | F9 |  | 1 |  | MN709486 |
| P17E11_TZ | F8 |  | 1 |  | MN709485 |
| P13A10_TZ | F14 |  | 1 |  | MN709488 |
| P6G7_UG | F4 | F8 | 1 | 1 | MN709433 |
| P2E8_TZ | F3 |  | 1 |  | MN709418 |
| P3F12_UG |  | F1, F2, F6, F12 |  | 6 | MN709410 |
| P2H2_TZ* | F2 |  | 1 |  | MN709490 |
| P4G11_TZ* | F7 |  | 1 |  | MN709495 |
| P5F5_TZ* | F7 |  | 1 |  | MN709494 |
| P6C3_TZ* | F7 |  | 1 |  | MN709492 |
| P5G5_TZ* | F7 |  | 1 |  | MN709493 |
| P4F11_TZ* | F7 |  | 1 |  | MN709496 |
| P5C11_TZ* | F7 |  | 2 |  | MN709491 |
| P3E4_TZ* | F4 |  | 4 |  | MN709414 |

A total of 1070 adult *B. tabaci* sampled from different fields in Tanzania and Uganda were analysed. Some of the species belong to unidentified species (^*^), with the closest species found at 85.1% identity from *B. afer* and 94.3% with SSA10.
